# Developmental dynamics of two bipotent thymic epithelial progenitor types

**DOI:** 10.1038/s41586-022-04752-8

**Published:** 2022-05-25

**Authors:** Anja Nusser, Sagar, Jeremy B. Swann, Brigitte Krauth, Dagmar Diekhoff, Lesly Calderon, Christiane Happe, Dominic Grün, Thomas Boehm

**Affiliations:** 1 Department of Developmental Immunology, Max Planck Institute of Immunobiology and Epigenetics, Freiburg, Germany; 2 Quantitative Single Cell Biology Group, Max Planck Institute of Immunobiology and Epigenetics, Freiburg, Germany; 3 Department of Medicine II, University Hospital Freiburg, Freiburg, Germany; 4 Würzburg Institute of Systems Immunology, Max Planck Research Group at the Julius-Maximilians-Universität Würzburg, Würzburg, Germany; 5 Helmholtz Institute for RNA-Based Infection Research (HIRI), Helmholtz Centre for Infection Research (HZI), Würzburg, Germany; 6 Faculty of Medicine, University of Freiburg, Freiburg, Germany; 7 Present Address: Institute of Molecular Pathology, Vienna, Austria

**Keywords:** Immunology, Cell biology

## Abstract

T cell development in the thymus is essential for cellular immunity and depends on the organotypic thymic epithelial microenvironment. In comparison with other organs, the size and cellular composition of the thymus are unusually dynamic, as exemplified by rapid growth and high T cell output during early stages of development, followed by a gradual loss of functional thymic epithelial cells and diminished naive T cell production with age^1–10^. Single-cell RNA sequencing (scRNA-seq) has uncovered an unexpected heterogeneity of cell types in the thymic epithelium of young and aged adult mice^11–18^; however, the identities and developmental dynamics of putative pre- and postnatal epithelial progenitors have remained unresolved^1,12,16,17,19–27^. Here we combine scRNA-seq and a new CRISPR–Cas9-based cellular barcoding system in mice to determine qualitative and quantitative changes in the thymic epithelium over time. This dual approach enabled us to identify two principal progenitor populations: an early bipotent progenitor type biased towards cortical epithelium and a postnatal bipotent progenitor population biased towards medullary epithelium. We further demonstrate that continuous autocrine provision of Fgf7 leads to sustained expansion of thymic microenvironments without exhausting the epithelial progenitor pools, suggesting a strategy to modulate the extent of thymopoietic activity.

## Abstract (web summary)

Two populations of thymic progenitors are identified, an early bipotent progenitor type biased towards cortical epithelium and a postnatal bipotent progenitor type biased towards medullary epithelium.

## Main

Differentiation of thymic epithelial cells (TECs) is dependent on the Foxn1 transcription factor^28–35^, and defects in epithelial specification and development are known to block T cell development, resulting in profound immunodeficiency and/or autoimmunity^36,37^. Because the thymic epithelium occupies such a central role in the formation and maintenance of cellular immunity, it has become an attractive target for immunomodulatory and regenerative therapies^38–43^ designed to correct congenital lack or iatrogenic loss of thymic tissue or to modify failing central tolerance. However, despite the immunological importance of TECs, central aspects of the biology of these cells remain unresolved. Progenitor activity in the embryonic thymus is associated with cells expressing *Psmb11*, encoding a thymus-specific component of the immunoproteasome^1,24,25^; however, the presence of a bipotent epithelial progenitor (or multiple bipotent progenitors)^22,23^ capable of giving rise to the cortical and medullary regions of the adult thymus, as well as the many different specialized epithelial subtypes^11–18^, has not yet been demonstrated. Here a high-resolution CRISPR–Cas9-based barcoding scheme combined with single-cell RNA sequencing (scRNA-seq) identifies bipotent progenitors in embryonic and adult TEC populations and shows their developmental relationship. Notably, we also demonstrate that continuous signalling via Fgfr2b causes a massive and sustained quantitative increase in TEC numbers, without altering the dynamic qualitative changes associated with the ageing thymic microenvironment.

## Cellular heterogeneity among TECs

**Fig. 1:**
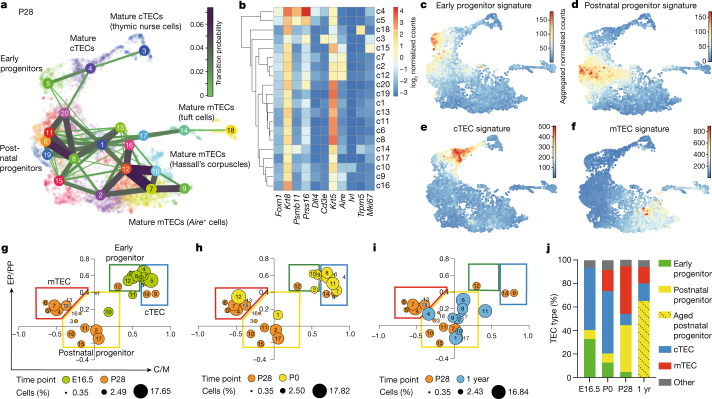
Heterogeneity of TECs. **a**, UMAP representation of transcriptome similarities among 6,959 individual TECs derived from 4-week-old wild-type male (*n* = 2) and female (*n* = 2) mice. Cell clusters and transition probabilities were inferred with VarID^44^; connections with probability *P* > 0.001 are shown, with transition probabilities indicated by line thickness and colour. The positions of clusters containing early and postnatal bipotent progenitors and mature cTEC and mTEC clusters are indicated. Colours mark cells in the identified cell clusters. **b**, Expression profiles of signature genes in individual TEC clusters. **c**–**f**, UMAP plots highlighting the aggregated expression profiles of gene groups distinguishing early (**c**) and postnatal (**d**) progenitors and cTECs (**e**) and mTECs (**f**). **g**–**i**, Age-dependent changes in the TEC compartment. Transcriptome features of TEC clusters are shown at various time points expressed as ratios of progenitor and mature TEC gene set transcript counts; the P28 time point was used as a reference. Assignment of clusters to the four main populations in the coordinate system is indicated in **g**; the sizes of dots correspond to the relative fraction in the TEC population. **j**, Summary of dynamic changes in the composition of the TEC compartment. yr, year. Source data

**Extended Data Fig. 1:**
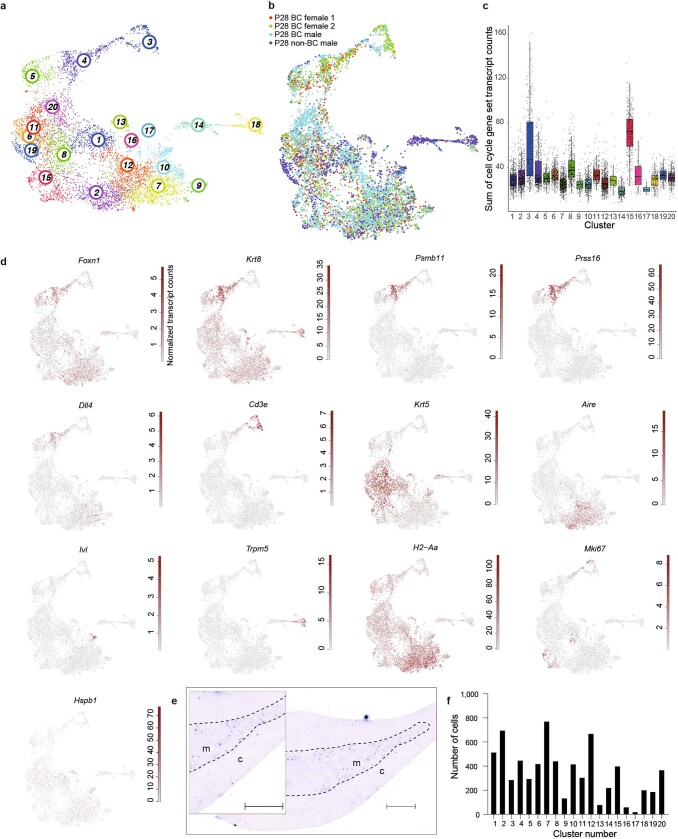
Characterization of TECs isolated from 4 week-old mice. **a**, UMAP representation highlighting the 20 clusters identified by VarID. **b**, Distribution of male and female TECs (colour-coded) in the UMAP map. **c**, Expression levels of cell cycle-related genes (see Supplementary Table 5). Cluster 3 corresponds to thymic nurse cells, which have a mixed phenotype of cTECs and thymocytes, the latter likely contributing to the proliferative signature; cluster 15 has affinities to the postnatal progenitor cluster but already expresses significant levels of *Aire*, a marker of mature mTECs, suggesting that this cluster harbours transit amplifying cells feeding into the mature mTEC populations. The first and third quartiles are marked by the box, the median is denoted by a horizontal line, the boundaries of the whiskers are set at 1.5 times the interquartile range, outliers are indicated as dots outside the boundary of the whiskers. Numbers of cells are as follows: Cluster 1, n = 516; cluster 2, n = 697; cluster 3, n = 288; cluster 4, n = 449; cluster 5, n = 296; cluster 6, n = 420; cluster 7, n = 773; cluster 8, n = 443; cluster 9, n = 136; cluster 10, n = 418; cluster 11, n = 306; cluster 12, n = 670; cluster 13, n = 81; cluster 14, n = 222; cluster 15, n = 401; cluster 16, n = 61; cluster 17, n = 21; cluster 18, n = 203; cluster 19, n = 189; cluster 20, n = 369. **d**, Expression profiles of the indicated signature genes. **e**, RNA *in situ* hybridization depicting *Hspb1* expression patterns in the thymus of 4-week-old mice; cortex (c) and medulla (m) are indicated, dashed lines highlight the cortico-medullary junction. *Hspb1*, as well as other genes not shown here, are often referred to as stress-related genes that may become upregulated during tissue dissociation and single-cell isolation, causing artefactual associations in the transcriptome analysis^78^. However, when assayed by RNA *in situ* hybridization in the intact thymic lobe of 4-week-old mice, *Hspb1* marks a subset of medullary cells, indicating that its expression is an intrinsic characteristic of TECs and confirming that its expression profile deduced from scRNA-seq (see panel d) is not affected by the isolation procedure. Scale bar, 0.5 mm. **f**, Number of cells in individual TEC clusters. Cluster 17 represents cells derived from ectopic parathyroid tissue Source data

We used scRNA-seq by CEL-Seq2 (refs. ^44,45^) to examine the cellular heterogeneity of CD45^–^EpCAM^+^ TECs from 4-week-old (postnatal day (P) 28) mice (Extended Data Fig. 1a, b). Cells with similar transcriptional profiles were identified by Louvain clustering using VarID^46^, and their predicted relationships were quantified by VarID transition probabilities. In agreement with previous studies^1,11–18^, the resulting Uniform Manifold Approximation and Projection (UMAP) plot (Fig. 1a) illustrates the complexity of the epithelial compartment in terms of gene expression profiles and cluster sizes (Fig. 1b and Extended Data Fig. 1c–f). At this age, few sex-related differences were apparent (Extended Data Fig. 1a, b).

## Identification of putative progenitors

We next sought to identify candidate progenitor populations within the epithelial compartment. Cells in several of the transcriptionally defined clusters expressed genes associated with mature TECs, including medullary TECs (mTECs; *Aire* and *Ivl*), tuft cells (*Trpm5*), cortical TECs (cTECs; *Prss16*) and nurse cells (*Prss16* and *Cd3e* co-expression, indicative of cTECs with enclosed thymocytes^47^), and were therefore excluded from our search, as mature TECs are unlikely to possess progenitor potential. Furthermore, we excluded highly proliferative cells (expressing *Mki67*) and those lacking expression of *Foxn1*, which is known to be expressed in TEC progenitor cells^22,34^ (Fig. 1b and Extended Data Fig. 1c–e). We then considered the transition probabilities (links) between the eight remaining candidate progenitor clusters (c1, c5, c6, c8, c11, c13, c19 and c20). Cluster c5 had links to mature cTECs (c3 and c4) and to c1 and c20, of which the latter two expressed *Krt5*, a marker of the mTEC lineage. Except for mature cTECs in c4, c5 exhibited the highest level of *Psmb11* expression, which is indicative of mature cTECs^48^ but also cells possessing progenitor potential, at least during embryogenesis^1,24,25^ and in the early postnatal period^49^. Hence, c5 exhibited features consistent with a bipotent progenitor. The transcriptomes of c6, c11 and c19 were very similar and had affinity for c1, which itself was connected to c5, c8 and c13 (Fig. 1b and Extended Data Fig. 1d). Cells in c6, c8, c11, c13 and c19 expressed *Krt5* but only low levels of *Psmb11*, in line with the view that, in contrast to the situation in the embryo^24^, adult mTECs do not directly originate from a *Psmb11*-expressing TEC compartment^1,25^. Collectively, these analyses suggest the presence of at least two potential bipotent progenitor cell types: one progenitor population exhibiting a bias towards cTEC development (represented by c5; henceforth referred to as ‘early progenitors’) and another more heterogeneous progenitor population exhibiting a distinct mTEC bias (represented by c1 and c6; henceforth referred to as ‘postnatal progenitors’).

## Age-dependent dynamics of TEC populations

**Extended Data Fig. 2:**
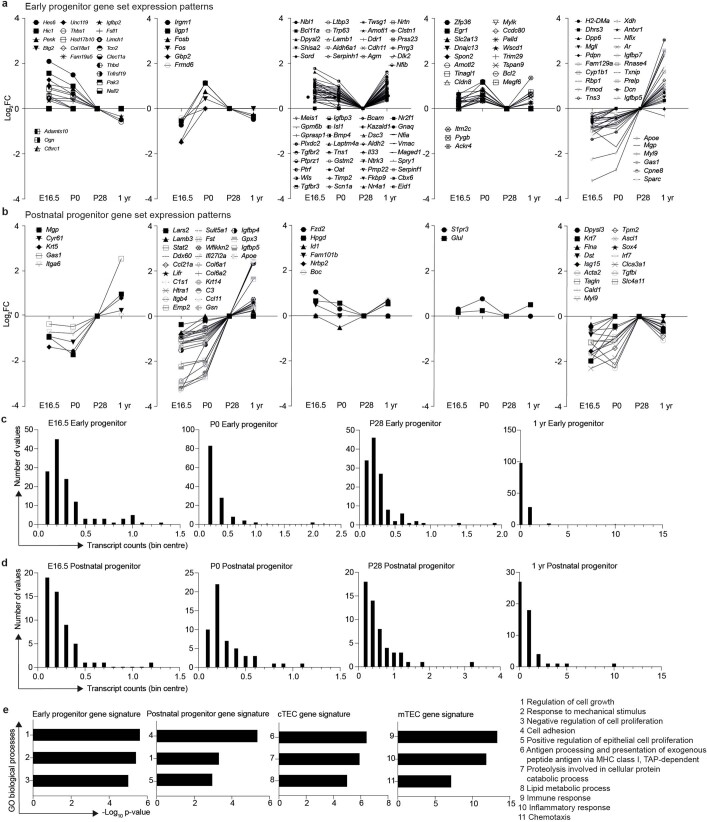
Characterization of gene sets defining embryonic and postnatal progenitors. **a, b**, Five transcriptional trajectories each define the gene sets characterizing embryonic (**a**) and postnatal (**b**) progenitors. Gene lists are given in Supplementary Tables 1 and 2. The P28 time point is used as the reference point for expression levels. **c**, **d**, Expression values of individual genes in the sets characterizing embryonic (**c**) and postnatal (**d**) progenitors. Most genes exhibit low expression values. **e**, Pathway analysis of unique gene sets (Supplementary Tables 1–4) characterizing early and postnatal progenitors and mature cTECs and mTECs. The three most enriched biological processes each as defined by the database for annotation, visualization and integrated discovery (DAVID) annotation tool^75,76^ are shown; the genes driving the enrichment for the GO categories are listed in Supplementary Table 6. Both progenitor populations express a number of heat shock protein genes, which are not considered here Source data

**Extended Data Fig. 3:**
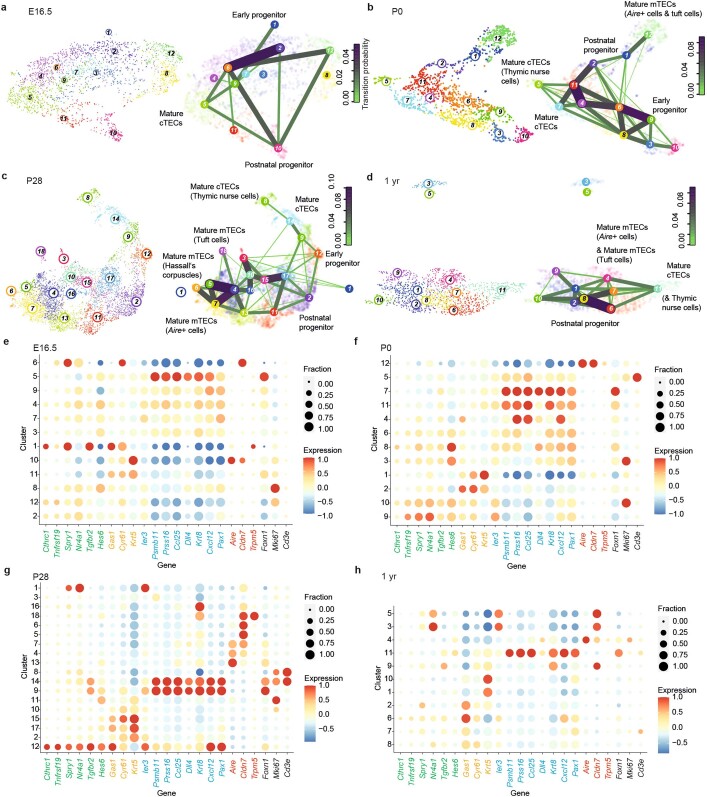
Age-dependent changes in the TEC compartment. **a–d**, UMAP representation of transcriptome similarities between individual TECs isolated from thymi at various time points. Note that for P28, a combined analysis of three mice (2 females, 1 male) is shown, as they were subsequently used for the barcoding analysis; in Fig. 1, data from an additional non-barcoded mouse is included. Left panels in a-d indicate the cluster designation deduced by VarID. The right panels indicate the transcriptional relationships in terms of VarID-derived transition probabilities; connections with probabilities P > 0.001 are shown and the transition probabilities are indicated by line thickness and colouring. For orientation purposes, the major cell populations are also indicated. Cells derived from ectopic parathyroid tissue were detected at P28 (**c**, cluster 8), and 1 year (**d**, clusters 3 and 5). **e–h**, Expression profiles of TEC clusters for the indicated signature genes and the four time points. The fractions of each cluster expressing a particular gene and their respective expression levels are depicted according to the scales shown on the right. Dot colour represents the z-score of the mean expression levels of the gene in the respective cluster, and dot size represents the fraction of cells in the cluster expressing the gene; gene names are coloured according to shared expression patterns (EP: green; PP: orange; cTEC, blue; mTEC, red; other genes of interest, black). z-scores above 1 and below −2 are replaced by 1 and -1 respectively.

**Extended Data Fig. 4:**
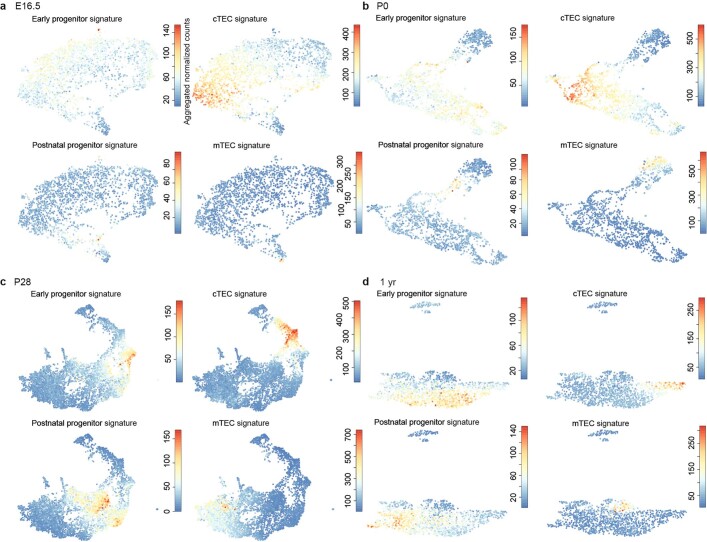
Age-dependent changes in the TEC compartment. **a–d**, UMAP maps of progenitor and mature TEC populations at 4 different time points; embryonic day 16.5 (E16.5), newborn (P0), 4-week-old (P28), and 1-year-old (1 yr). The UMAP maps for P28 mice were generated by inclusion of only barcoded mice.

**Extended Data Fig. 5:**
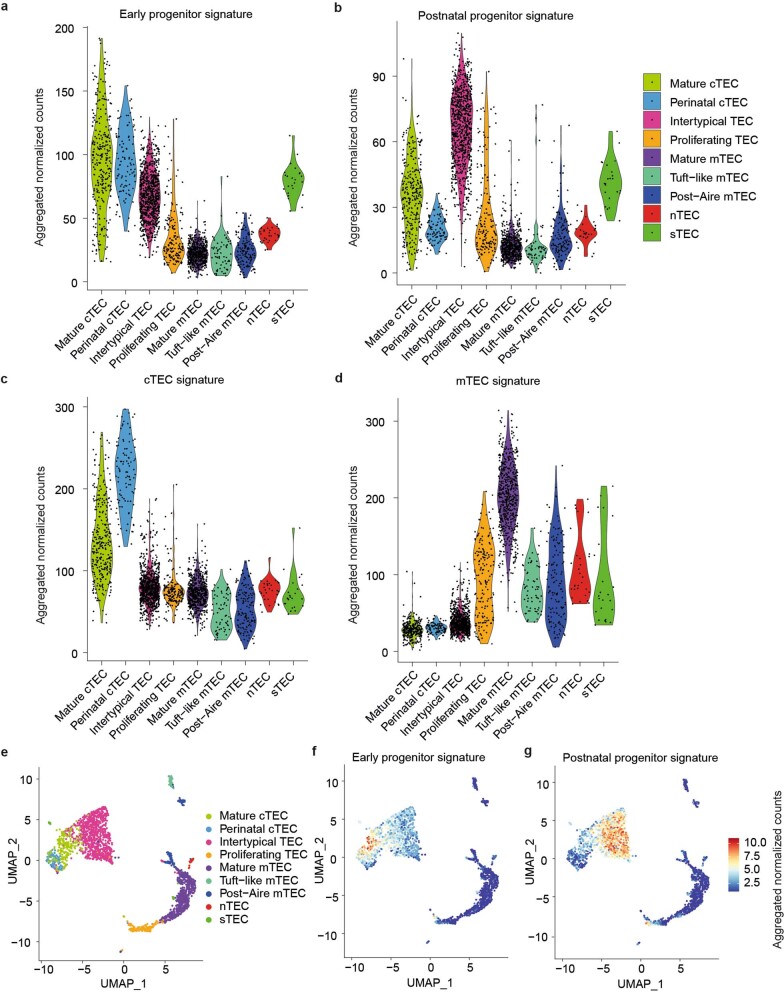
Comparative analysis of scRNA-seq data. **a–d**, Projection of aggregated read-counts for gene sets defining early and postnatal progenitors and mature cTEC and mTEC (Supplementary Tables 1–4) onto the 9 major TEC subsets defined by Baran-Gale *et al*. (*1*). Whereas the early progenitor signature cannot be unequivocally assigned, the postnatal progenitor signature maps to the intertypical TEC subset; the cTEC and mTEC signatures match the description of Baran-Gale *et al*.^1^. **e**, Analysis of scRNA-seq data of Baran-Gale *et al*.^1^ visualized using UMAP. Their 9 different TEC subtypes are distinguished by different colours, matching the code in a-d. **f**, Projection of aggregated read-counts for the early progenitor gene set onto the UMAP, indicating partial overlap with cells referred to as mature and perinatal cTECs. **g**, Projection of aggregated read-counts for the postnatal progenitor gene set onto the UMAP indicating good correspondence with the majority of intertypical TECs.

Given the presumed developmental dynamics of TEC progenitors, we tested the hypothesis that the early progenitor population dominates in the embryonic and perinatal stages of development, whereas the postnatal progenitor population is more prevalent in adolescent and adult stages. To do this, we assigned four largely non-overlapping gene sets to mark the two progenitor populations (Supplementary Tables 1 and 2, and Extended Data Fig. 2a, b) and the mature cTEC and mTEC populations (Supplementary Tables 3 and 4). Population-specific scores were calculated by summation of transcript counts in the four separate gene lists. Notably, although the genes in these sets showed different temporal dynamics, the aggregated scores were not dominated by individual highly expressed genes (for example, see Extended Data Fig. 2c, d); pathway analysis associated regulation of cell growth with the two progenitor populations and immune-related processes with the two mature TEC populations (Extended Data Fig. 2e). The aggregated expression levels of progenitor and mature TEC gene sets mark four distinct domains in the UMAP plot of 4-week-old (P28) mice (Fig. 1c–f). At embryonic day (E) 16.5, the transcriptional landscape of TECs was dominated by the cTEC signature and early progenitor cells (Extended Data Figs. 3a, e and 4a). In new-born mice (P0), the number of postnatal progenitors and mTECs began to increase (Extended Data Figs. 3b, f and 4b). At P28, cells with the postnatal progenitor signature were more numerous than those with the early progenitor signature; moreover, the cTEC compartment was much smaller than at earlier stages, with mTEC-like cells dominating the TEC population (Extended Data Figs. 3c, g and 4c). At 1 year of age, the TEC compartment exhibited signs of functional deterioration. At this time, mature cTECs and mTECs made up only a small fraction of the thymic epithelia; by contrast, an unusually large number of cells simultaneously exhibited signatures of both progenitor types. These features suggest that, in aged mice, expanded progenitor-like cells may have lost their defining characteristics and that this indistinct phenotype is associated with low differentiation potential of these aged progenitors (Extended Data Figs. 3d, h and 4d). In a previous study, a putative progenitor population was identified with a distinct mTEC bias^1^; on the basis of gene expression profiles, the ‘intertypical’ TECs described in the study are closely related to the postnatal progenitor population defined here (Extended Data Fig. 5).

To visualize dynamic age-related changes in the TEC compartment, we calculated the ratios of transcript scores for early and postnatal progenitors (EP/PP) and for mature cTEC and mTEC populations (C/M) for each cell cluster and plotted them with reference to the P28 time point (Fig. 1g–i). Whereas the E16.5 TEC compartment was dominated by cells closely resembling early progenitors and mature cTECs (Fig. 1g), the P0 time point reflected the transition from an embryonic to an adult TEC compartment, as exemplified by the composition at P28 (Fig. 1h). The 1-year time point was characterized by few mature TECs (Fig. 1i). At this stage, most cell clusters populating the postnatal progenitor compartment (c2, c6, c7, c8 and c9) exhibited increased EP/PP ratios when compared with the corresponding P28 cell clusters, as a result of increased expression levels of genes that are associated with early progenitors; we refer to TECs with this indistinct phenotype as ‘aged progenitors’ (Fig. 1i, j). Our results support the notion that progenitor compartment(s) increase with age^1^. The age-dependent differences in TEC composition are summarized in Fig. 1j.

## Shared ancestry of Ly51^+^ and UEA-1^+^ TECs

**Fig. 2:**
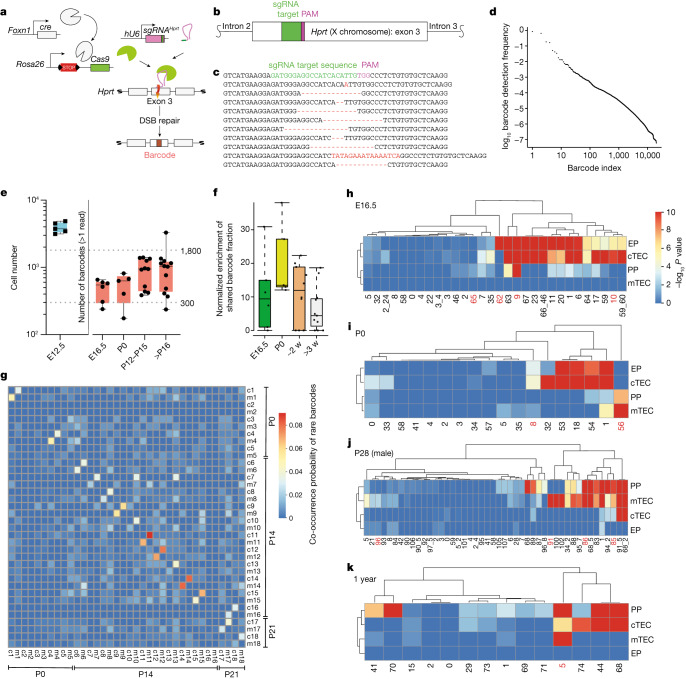
Barcoding shows the differentiation capacity of progenitor populations. **a**, Schematic of the CRISPR–Cas9-based barcoding system. DSB, double-strand break. **b**, Location of the target site in exon 3 of the mouse *Hprt* gene. **c**, Examples of barcodes; the germline sequence is shown with the sgRNA target and protospacer adjacent motif (PAM) sequences indicated at top. Nucleotide additions and deletions (dashes) are indicated in red. **d**, Frequencies of individual barcodes in decreasing order. **e**, Number of *Foxn1*-expressing TECs in the thymic rudiment of E12.5 embryos^9^ (left; *n* = 5) and numbers of different barcodes in the thymi of mice of different ages (right): E16.5, *n* = 6; P0, *n* = 5; P12–P15, *n* = 11; >P16, *n* = 12. The dotted lines indicate the range of the numbers of progenitors previously inferred from medullary islet counts in adult mice^26^. **f**, Enrichment of shared barcodes in the Ly51^–^UEA-1^+^ mTEC and Ly51^+^UEA-1^–^ cTEC fractions of mice of different ages. Enrichment values were significantly different in the comparison of mice at P0 and >3 weeks (w) (*P* = 0.009, one-sided Wilcoxon test). E16.5, *n* = 6; P0, *n* = 5; ~2 weeks, *n* = 11; >3 weeks, *n* = 11. For **e** and **f**, boxes extend from the 25th to 75th percentile; whiskers extend to the largest and smallest values; and the median is indicated. See the Methods for a definition of the enrichment value *E*_*m*_. **g**, Co-occurrence probability of rare barcodes across pairs of samples highlighting enhanced co-occurrence in mTEC (m) and cTEC (c) fractions of the same mouse; individual mice are identified by number. Data are shown for *n* = 18 mice. **h**–**k**, *P* values (–log_10_) of barcode frequencies indicating co-occurrence of individual barcodes in progenitor and mature TEC fractions (as defined in Fig. 1c–f) at different time points. For **g**–**k**, *P* values were calculated as described in the Methods and corrected for multiple testing by the Benjamini–Hochberg method. The red numbers refer to clones discussed in the text. Source data

**Extended Data Fig. 6:**
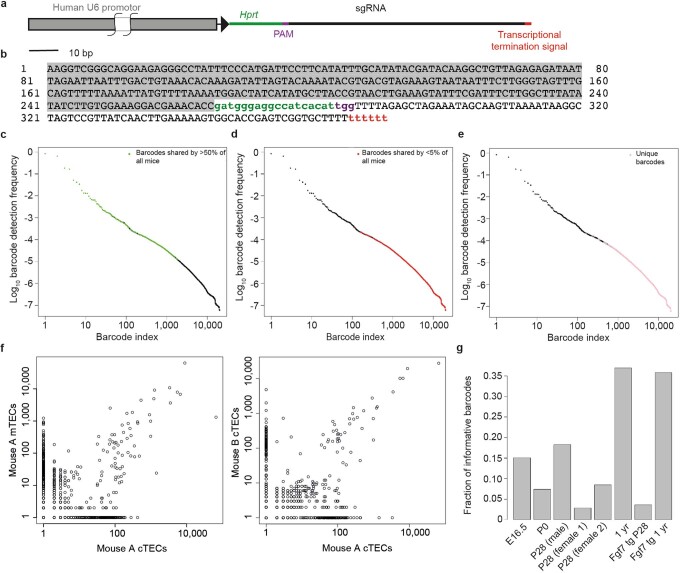
Characterization of the *sgRNA*^Hprt^ cassette. **a**, Schematic of the components of the *hU6:sgRNA*^Hprt^ transgene; key features are indicated by name and are colour-coded; the bar representing the hU6 promotor sequence was truncated. **b**, Nucleotide sequence of the *hU6:sgRNA*^Hprt^ transgene construct (colour code as in **a**). **c**–**e**, Frequencies of individual barcodes in decreasing order from left to right grouped by the degree of occurrence in the cohort of mice analysed here (n = 33); colours indicate those barcodes that satisfy the criterion indicated at the top right of each plot. **f**, Scatter plots of barcode frequencies for mTECs and cTECs from the same mouse versus barcode frequencies in cTECs isolated from two different mice. **g**, Fraction of informative barcodes observed in the TEC compartment of individual mice; informative barcodes are those whose *P* values indicate a significant deviation (*P*_*b,i*_ < 0.001 for barcode *b* in sample *i*) from the barcode frequencies expected from the background model. Since these values represent singular data points, statistical comparisons were not done. Barcode data are listed in Supplementary Tables 7–14 Source data

**Extended Data Fig. 7:**
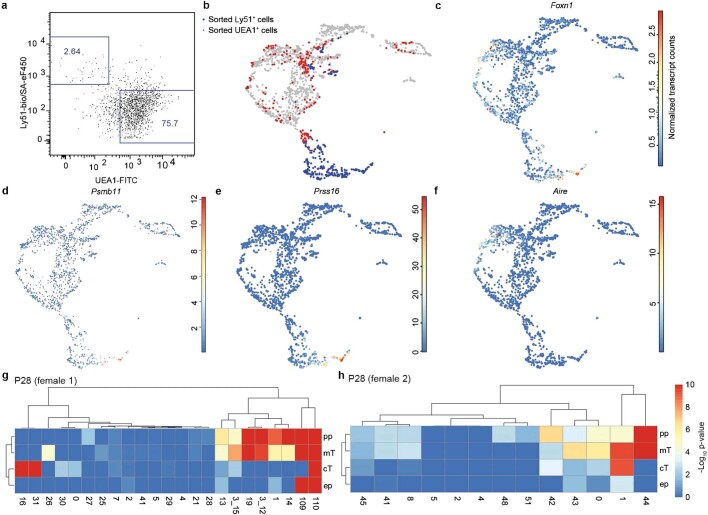
Characterization of wild-type TEC subsets at P28. **a**, Flow cytometric analysis of EpCAM^+^CD45^–^ TECs after co-staining with anti-Ly51 antibody and UEA-1 lectin. The cTEC gate is indicated on the upper left (2.64% of TECs), the mTEC gate is indicated on the lower right (75.7% of TECs). **b**, UMAP representation of transcriptome similarities resulting from the combined analysis of three types of wild-type TECs; EpCAM^+^ TECs without further purification, and TECs purified according to positive Ly51 and UEA-1 staining characteristics, with origins of TECs indicated by colours (EpCAM^+^ TECs without further purification, grey; Ly51^+^, blue; UEA-1^+^, red). **c–f**, Gene expression profiles of *Foxn1* (c), *Psmb11* (d), *Prss16* (e), and *Aire* (f), depicted as normalized absolute counts. **g**, **h**, Reproducibility of lineage relationships in barcoded TEC populations of female mice; see Fig. 2j for the pattern of the male mouse. P values were calculated as described in the Methods section and multiple-testing corrected by the Benjamini-Hochberg method.

To further explore potential progenitor–progeny relationships in the TEC compartment, we developed a high-resolution lineage tracing method based on CRISPR–Cas9-mediated scarring in exon 3 of the *Hprt* gene (Fig. 2a, b and Extended Data Fig. 6a, b). In the *hU6-sgRNA*^*Hprt*^; *Foxn1-cre*; *Rosa26-*flox-STOP-flox-*Cas9* triple-transgenic mice used here, TECs are marked in early embryogenesis as soon as *Foxn1* expression begins at around E11.5 (ref. ^50^). Because essentially all embryonic and adult TECs have a history of *Foxn1* expression^34^, the scars (Fig. 2c) introduced in individual epithelial cells of the thymic rudiment in early embryogenesis indelibly mark their subsequent progeny. As is the case with other barcoding schemes^51,52^, individual scars, referred to as barcodes below, are generated at different frequencies (Fig. 2d); the most frequent sequences were shared by different mice (Extended Data Fig. 6c–e). The total number of different barcodes per thymus was on the order of 500–1,000 (Fig. 2e), close to the number of medullary islets observed in adult mice (300–1,800)^26^. We found a significant enrichment (Extended Data Fig. 6f) of barcodes that were shared by the EpCAM^+^CD45^–^Ly51^+^UEA-1^–^ cTEC and EpCAM^+^CD45^–^Ly51^–^UEA-1^+^ mTEC subsets (Extended Data Fig. 7a–f) of male mice, at several pre- and postnatal time points (Fig. 2f), suggesting a common origin for these subsets. We then identified barcodes that were represented only twice in the purified TEC populations of all mice, referred to as rare barcodes for the purpose of this experiment, and determined the probabilities of their co-occurrence in cTECs and mTECs of the same mouse versus any other mouse. On average, the corresponding mTEC and cTEC samples from the same mouse shared 3.5% of rare barcodes, whereas samples from different mice shared only 0.27% of rare barcodes. Without prefiltering based on barcode frequency, the fraction of barcodes shared by mTEC and cTEC populations was >50%. The significant degree of co-occurrence of such rare barcodes in mTEC and cTEC samples from the same mouse (Fig. 2g) suggests that cTECs and mTECs have a shared ancestor.

## Combining scRNA-seq and barcode tracing

**Extended Data Fig. 8:**
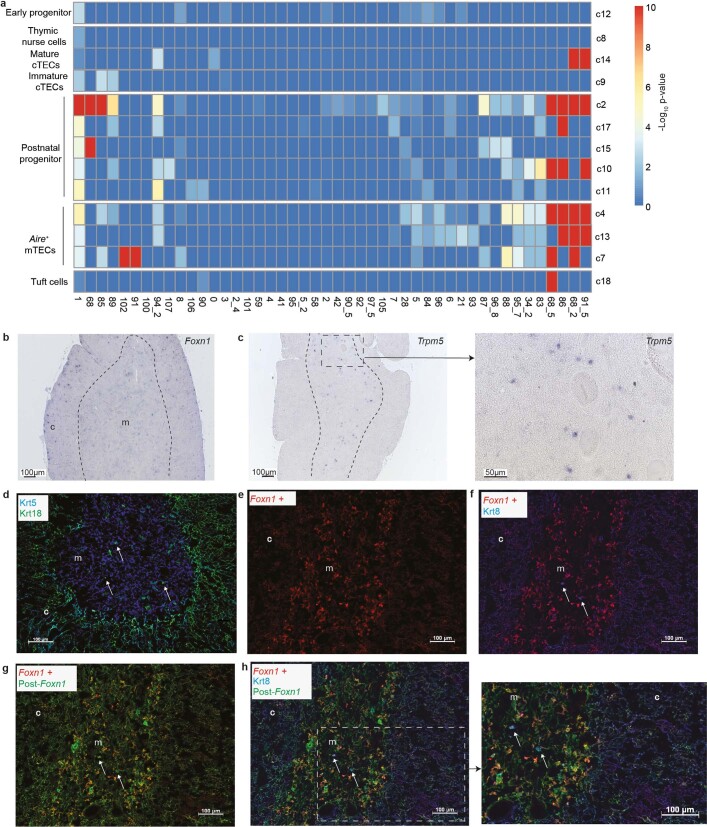
Characterization of clonal relationships in the mTEC compartment. **a**, Co-occurrence of individual barcodes in individual TEC clusters (as defined in Extended Data Fig. 3c; 4c) at P28; the -log_10_ *P* values of barcode frequencies are indicated. *Aire*-positive cells share several barcodes with the postnatal progenitor population but also exhibit private barcodes (barcodes 91 and 102); this observation may be explained by the fact that a certain progenitor originally giving rise to the *Aire*-expressing cells has ceased to exist; alternatively, sublineage-restricted progenitors and their descendants that appear with developmental time may at some point outnumber the original bipotent ancestor, resulting in a lower sampling probability of the latter. *Trmp5*-expressing tuft cells^13,15^ (cluster18) share barcodes 1 and 68_5 with the postnatal progenitor population and the *Aire*^+^ mTEC compartment, suggesting that they belong to the mTEC lineage. *P* values were calculated as described in the Methods section and multiple-testing corrected by the Benjamini-Hochberg method. **b**, **c**, Expression of *Foxn1* (left panel) and *Trmp5* (middle and right panels) genes was detected by RNA *in situ* hybridization using thymus sections of a 4- week-old wild-type mouse; the cortico-medullary junction is indicated by the dashed line; part of the medullary area (boxed) is shown as higher power view. Note that *Foxn1*-positive cells are present in both cortex and medulla, whereas *Trmp5*-expresing cells are found in the medulla only. *Trmp5*-positive cells express Krt8, but neither *Ivl* nor *Foxn1*; *Ivl*-expressing cells are Krt8 negative (Fig. 1b). **d**, Identification of scattered Krt18-positive cells in the medulla; most medullary cells express Krt5 (blue) and cortical cells express Krt18 (green). **e**, Active *Foxn1* expression as revealed by the activity of the *Foxn1:mCardinal* transgene using an anti-RFP antibody (red). **f**, *Foxn1* expression (as in **e**) in cortical and medullary TECs relative to Krt8 expressing TECs (anti-Krt8 antibody, blue); note that Krt8 typically identifies cTEC (as does Krt18). The rare Krt8 expressing cells in the medulla do not express *Foxn1* (arrows). **g**, Identification of *Foxn1*-expressing cells (as in **e**), post-*Foxn1* cells. *Foxn1* expression is recorded via the *Foxn1:mCardinal* transgenic construct (anti-RFP antibody; red). Post-*Foxn1* cells are identified by *Foxn1*-activated indelible EYFP expression in the *Foxn1:Cre*; *Rosa26-LSL-EYFP* reporter background (anti-GFP antibody, green); note the presence of purely green cells (arrows), indicating that such cells have lost *Foxn1* expression. **h**, Combined analysis of all three cell states; Krt8-positive cells are post-*Foxn1* cells (arrows); a magnification of the indicated area is shown on the left. Collectively, these data suggest that the Krt8-positive post-*Foxn1* cells in the medulla are tuft cells. Scale bars, 0.1 mm. Panels in b and c are representative of 3 mice; panels in d-h are representative of 2 mice.

Next, we applied simultaneous scRNA-seq and barcode tracing to dissect progenitor–progeny relationships within the TEC compartments of mice of different ages (Fig. 2h–k). To gain insight into the distribution of individual barcodes, we compared the barcode frequencies in each compartment to the expected barcode frequencies obtained from the bulk samples of 33 mice (Fig. 2d and Extended Data Fig. 6g). At E16.5, about two-thirds of the barcode sequences were found in early progenitors; the majority of barcodes enriched in early progenitors were also over-represented in cTECs (for example, barcode 10) (Fig. 2h), confirming the notion^19,21^ that the early progenitor population has a distinct bias towards differentiation into cTECs. The presence of shared barcodes (for example, barcode 9) also indicated a developmental relationship between early and postnatal progenitor populations. Some early and postnatal progenitor cells had not yet contributed to cTECs or mTECs by this time in development (barcodes 62 and 65, respectively). At P0, the most notable additions to lineage relationships (Fig. 2i) concerned the presence of a postnatal progenitor giving rise predominantly to mTECs (barcode 56) and the presence of an embryonic progenitor giving rise to both cTECs and mTECs (barcode 8). At P28, cells with the transcriptional signature of early progenitors predominantly gave rise to mTECs (barcode 96) rather than both mTECs and cTECs; the number of postnatal progenitors biased towards mTEC differentiation (barcode 85,86) increased (Fig. 2j), a pattern that was independent of the sex of the animal (Extended Data Fig. 7g, h). Interestingly, several barcodes uniquely over-represented in mTECs were also observed (for example, barcode 91), suggesting the existence of compartment-specific progenitor activity (Extended Data Fig. 8); the corresponding barcodes may no longer be detectable in the bipotent progenitor populations either because the particular progenitor clones have ceased to exist or because they have too low a frequency to be reliably sampled. Finally, although the TEC compartment of aged mice lacked evidence of productive early progenitor cell types, bipotent progenitors were present (barcode 5) (Fig. 2k); however, most barcodes in aged postnatal progenitors were linked to cells with the cTEC signature, in line with the notion that cTEC-like cells increase in frequency in aged mice^2,9^. Collectively, our results illustrate the advantage gained from using a barcoding scheme in the identification and characterization of progenitor populations and their progeny when this approach is combined with transcriptome data at single-cell resolution.

## Fgf signalling does not exhaust bipotent progenitors

**Fig. 3:**
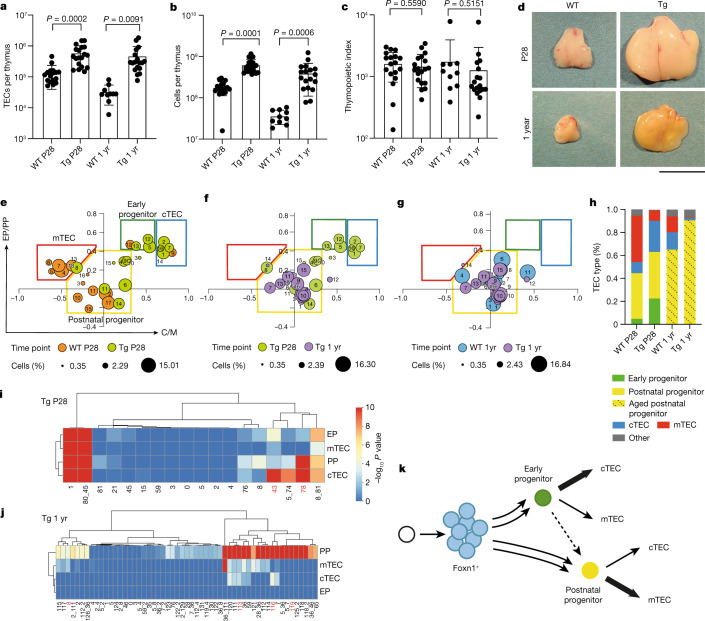
Autocrine Fgf stimulation results in sustained thymic hyperplasia. **a**–**c**, Quantitative assessment of thymopoiesis in wild-type (WT) and *Foxn1-Fgf7* transgenic (Tg) mice at 4 weeks and 1 year of age. **a**, WT P28, *n* = 18;Tg P28, *n* = 19; WT 1 yr, *n* = 10;Tg 1 yr, *n* = 18. **b**, WT P28, *n* = 19; Tg P28, *n* = 21; WT 1 yr, *n* = 10; Tg 1 yr, *n* = 18. **c**, WT P28, *n* = 18; Tg P28, *n* = 19; WT 1 yr, *n* = 10; Tg 1 yr, *n* = 18. Data are shown as the mean ± s.d. *P* values are indicated from two-sided *t* tests. **d**, Representative photographs of thymi from the mice analysed in **a**; scale bar, 10 mm. **e**–**g**, Transcriptome features of TEC clusters expressed as ratios of progenitor and mature TEC gene set transcript counts. Assignment of clusters to the four main populations in the coordinate system is indicated in **e**; the sizes of dots correspond to the relative fraction in the TEC population. **h**, Summary of dynamic changes in the composition of the TEC compartment. **i**, **j**, *P* values (–log_10_) of barcode frequencies indicating co-occurrence of individual barcodes in progenitor and mature TEC fractions (as defined in Fig. 1c–f) at two time points. *P* values were calculated as described in the Methods and corrected for multiple testing by the Benjamini–Hochberg method. The red numbers correspond to clones discussed in the text. **k**, Schematic indicating the divergent developmental trajectories of embryonic and postnatal epithelial progenitors. Line thickness corresponds to lineage bias; the dashed line indicates the presumptive lineage relationship of the two progenitor populations. Source data

**Extended Data Fig. 9:**
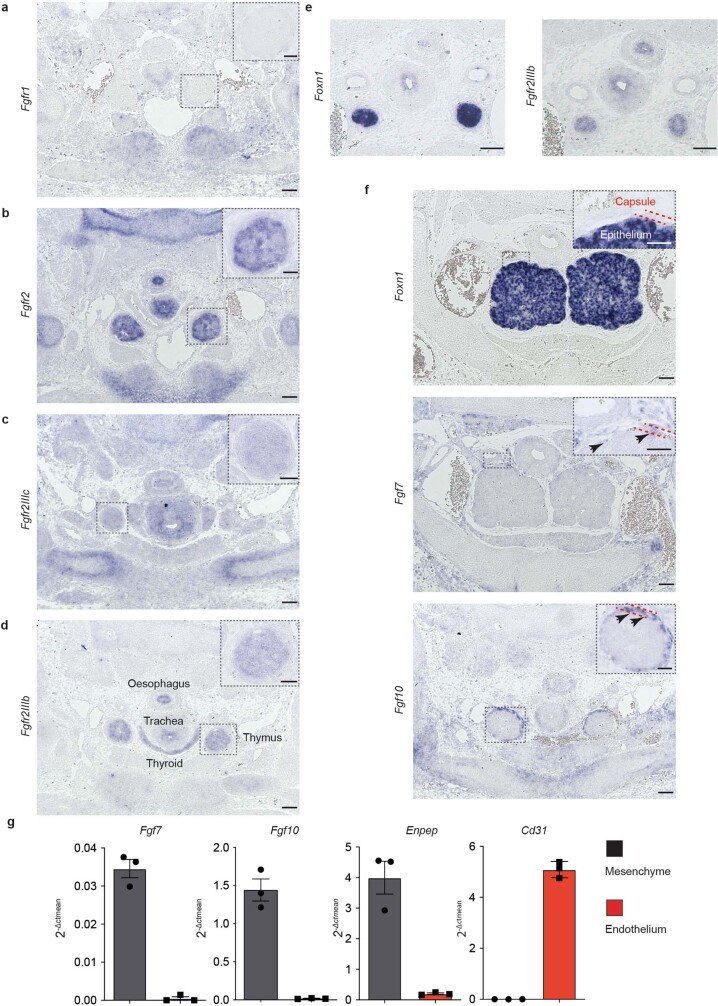
Characterization of the Fgfr2-signalling pathway in mouse embryos. **a–f**, RNA *in situ* hybridization (ISH) analysis of mouse embryos. **a–e**, ISH performed on E13.5 embryos indicates that *Fgfr2IIIb* (but neither *Fgfr2IIIc* nor *Fgfr1*) expression is a common feature of pharyngeal epithelia. **a**, No detectable expression of the *Fgfr1* gene in the thymic epithelium (higher magnification in inset). **b**, The *Fgfr2* gene is expressed in epithelia of pharyngeal organs, including the thymus (inset). **c**, Low levels of expression of *Fgfr2IIIc* in the thymus. **d**, Moderate levels of expression of *Fgfr2IIIb* in the thymus; anatomical structures are indicated. **e**, Expression of *Fgfr2IIIb* is present in E13.5 *Foxn1*-deficient thymic epithelial rudiment and thus independent of Foxn1 activity. **f**, Expression of *Fgf7* (E15.5, middle panel) and *Fgf10* (E13.5, bottom panel) genes in the mesenchymal capsule of the thymus (indicated by arrows), but not in the epithelium that is marked by *Foxn1* expression (E15.5, upper panel). The capsular zone is indicated with dashed red lines in the inset of each panel. **g**, qPCR analysis of gene expression patterns in purified thymic mesenchyme (isolated as CD45^–^EpCAM^–^CD31^–^Ly51^+^ cells) and endothelium (isolated as CD45^–^EpCAM^–^CD31^+^Ly51^–^ cells) of 4-week-old mice; data are shown as mean±s.e.m. n = 3 for all experiments. *Enpep* encodes the mesenchymal Ly51 marker (note that *Enpep* is also expressed on cTECs, which unlike mesenchymal cells also express the epithelial marker EpCAM); *Cd31* expression marks endothelial cells. This analysis indicates that of the many ligands of Fgfr2b^79,80^, Fgf7 and Fgf10 are expressed by thymic mesenchyme, but not endothelial cells. Embryo genotypes for **a**–**d**, *Foxn1*^+/−^, for **e**, *Foxn1*^−/−^; for **f** and **g** *Foxn1*^+/+^. Panels in a-f are representative of 3 mice. Scale bars, 0.1 mm for main panels; 0.05 mm for insets. Source data

**Extended Data Fig. 10:**
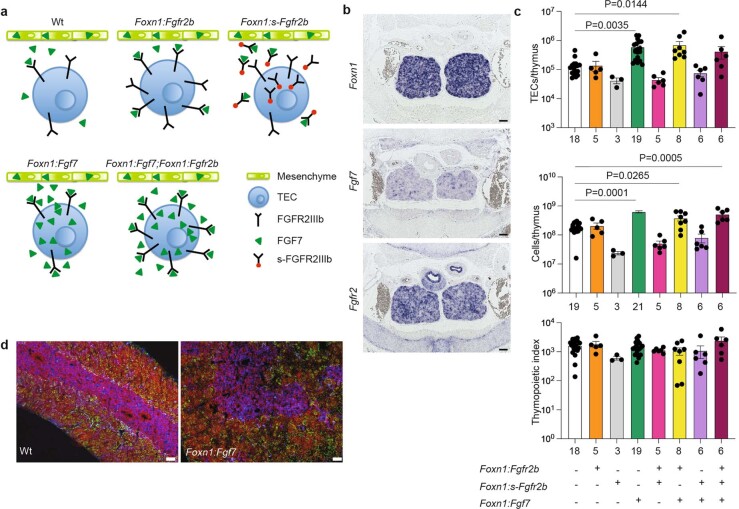
Strategy of transgenic interference targeting the Fgfr2-signalling pathway. **a**, Schematic illustration of the Fgf signalling pathway in the thymus. The mesenchyme (green) produces Fgf7 (and other Fgfr2 ligands) that bind to the Fgfr2IIIb variant of the receptor expressed by thymic epithelial cells (top row, left panel). In the present study, this pathway is genetically modulated in several ways to explore whether, under physiological conditions, the receptor or the ligand are in excess. We used the *Foxn1* promotor^63^ to direct expression of various components of the Fgf signalling cascade in thymic epithelial cells, either singly or in combination. (i) Overexpression of the Fgfr2b receptor (top row, middle panel). The *Foxn1:Fgfr2IIIb* transgenic is designed to increase the sensitivity of epithelial cells to Fgf ligands. This transgenic constellation should result in a stimulatory effect if the ligand is in excess, because more receptor/ligand complexes can form at the cell surface of the target cell. However, if the ligand rather than the receptor is limiting, providing more receptors should have no effect on the target cell. (ii) Expression of a soluble decoy receptor (top row, right panel). The *Foxn:s-Fgfr2IIIb* transgenic line expresses a soluble dominant-negative form of the Fgfr2IIIb receptor^60,81^ and is expected to disrupt productive Fgfr2IIIb receptor signalling. In this experiment, a decrease in the concentration of free ligand in the extracellular space should reduce engagement of receptors and thus diminish signalling activity. (iii) Autocrine provision of Fgf7 (bottom row, left panel). In the *Foxn1:Fgf7* transgenic line, an autocrine loop is generated in the thymic microenvironment by expression of Fgf7 in TECs, resulting in an increase in the concentration of free ligand in the extracellular space. If, in the wild-type situation, receptor molecules are in excess and hence mostly free of ligand, signalling activity should increase; conversely, if under normal circumstances the ligand is in excess over the receptor, no effect should be seen. (iv) Simultaneous overexpression of both Fgf7 ligand and Fgfr2IIIb receptor (bottom row, right panel). This constellation is designed to test if the effect of excess Fgf7 ligand can be further increased by provision of additional receptors; if so, it would indicate that endogenous receptors are fully occupied by excess free ligand. **b**, Overexpression of *Fgf7* (middle panel) and *Fgfr2IIIb* (bottom panel) in the thymic epithelium of *Foxn1:Fgf7-*transgenic and *Foxn1:Fgfr2IIIb*-transgenic mice respectively, as demonstrated by RNA *in situ* hybridization performed on sections from E15.5 embryos with the indicated probes. Scale bars, 0.1 mm. Panels are representative of 3 mice. **c**, Thymopoietic activities in 4-week-old mice of the indicated genotypes; the number of mice per genotype is indicated below each column; data are shown as mean±s.e.m. From multiple comparisons, only the statistically significant differences are indicated. No significant differences between male and female mice were observed; hence, data from animals of both sexes were pooled for the analysis. The variable extents of thymopoietic activity in the seven transgenic mouse lines studied herein indicate that, under physiological conditions, limiting levels of ligand(s) rather than those of the receptor determine the extent of Fgf signalling in TECs; note, for instance, that expression of a soluble Fgfr2IIIb decoy receptor impaired thymopoiesis in a wild-type background and even partially neutralized the autocrine effects of the *Foxn1:Fgf7* transgene. t-test; two-sided; multiple-testing corrected by Benjamini-Hochberg method. P values for significant differences (i. e., P < 0.05) are indicated. **d**, Immunohistochemical analysis of wild-type and *Foxn1:Fgf7* transgenic 4-week-old thymi; CD45-positive haematopoietic cells, red; Krt5-positive TECs, blue; Krt18-positive TECs, green. Scale bars, 0.1 mm. Panels are representative of 3 mice. Source data

**Extended Data Fig. 11:**
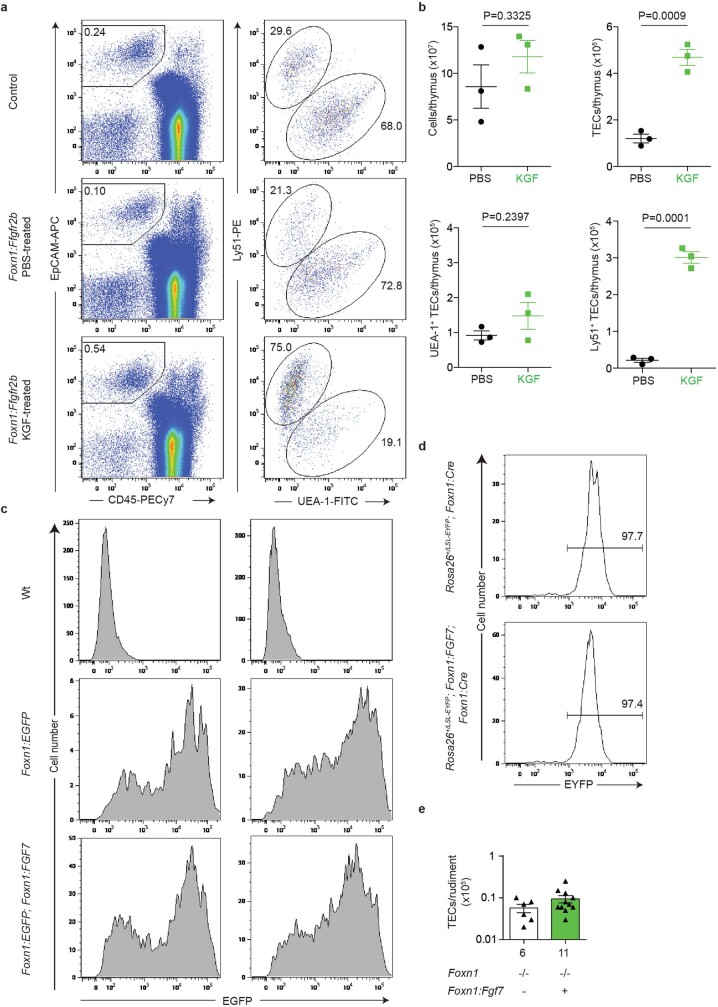
Response of the thymic epithelium to KGF-treatment and autocrine stimulation. These experiments are designed to test the hypothesis that acute provision of an Fgfr2b ligand increases the number of cells expressing the Fgfr2b receptor. **a**, Flow cytometric analysis of dissociated thymic tissue; the percentage of EpCAM^+^CD45^–^ TECs is indicated in the left panels. The cell surface pattern of TECs is resolved after co-staining with anti-Ly51 antibody and UEA-1 lectin (right panels). The control is from a 2-week-old wild-type thymus; representative profiles for *Foxn1:Fgfr2b* transgenic cohorts of male mice treated with PBS or KGF (human Fgf7) are shown in the middle and bottom rows, respectively. As shown in Extended Data Fig. 10, the presence of the transgene as such has no influence on the magnitude of the endogenous Fgf7 response. Unlike the response to continuous Fgf stimulation (c.f., Fig. 3), short-term treatment of adult mice with exogenous human Fgf7 (KGF) causes a disproportional increase in Ly51^+^ cells at the expense of UEA-1^+^ TECs. **b**, Numerical assessment of TEC subsets (n = 3 per condition; data are shown as mean±s.e.m.). At the age of four weeks, male mice received 9 intra-peritoneal injections of KGF at days 1, 2, 3, 8, 9, 10, 15, 16, 17 and were sacrificed on day 21; note the large increase of Ly51+ TECs after KGF treatment. The lack of a significant increase in thymocyte numbers indicates that the Fgf-responsive TEC compartment initially gives rise to functionally immature Ly51^+^ progeny^18^; this observation supports the notion that the Ly51^+^ TEC compartment is functionally heterogeneous and indicates that Ly51 expression as such does not unequivocally identify mature cTECs. t-test; two-sided; P values are indicated. **c**, Age-related down-regulation of *Foxn1*-expression in Fgf-stimulated TECs revealed by flow cytometry. Representative flow cytometric profiles of EpCAM^+^CD45^–^ TECs of 7 to 8 week-old female and male mice with the indicated genotypes; the non-transgenic wild-type cells serve as a negative control for reporter expression levels arising from the *Foxn1:EGFP* transgene^57^. Note that Fgf7 stimulation does not prevent the age-dependent physiological down-regulation of the *Foxn1* gene. **d**, *Foxn1*-negative TECs once expressed *Foxn1*, as indicated by the presence of indelible lineage marks in TECs of 4 to 6 week-old female mice (males show the same pattern) as revealed by the *Rosa26-LSL-EYFP; Foxn1:Cre* reporter line^58^. For **c** and **d**, the profiles are representative of at least 4 biological replicates. **e**, Fgf7 stimulation fails to increase the number of TECs in *Foxn1*-deficient mice. The numbers of mice in the two cohorts are shown below the histogram; data are shown as mean±s.e.m. Since *Foxn1*-deficient epithelia do not proliferate in response to Fgf stimulation, all changes in the TEC compartment described here are likely to originate from *Foxn1*-expressing cells. Source data

**Extended Data Fig. 12:**
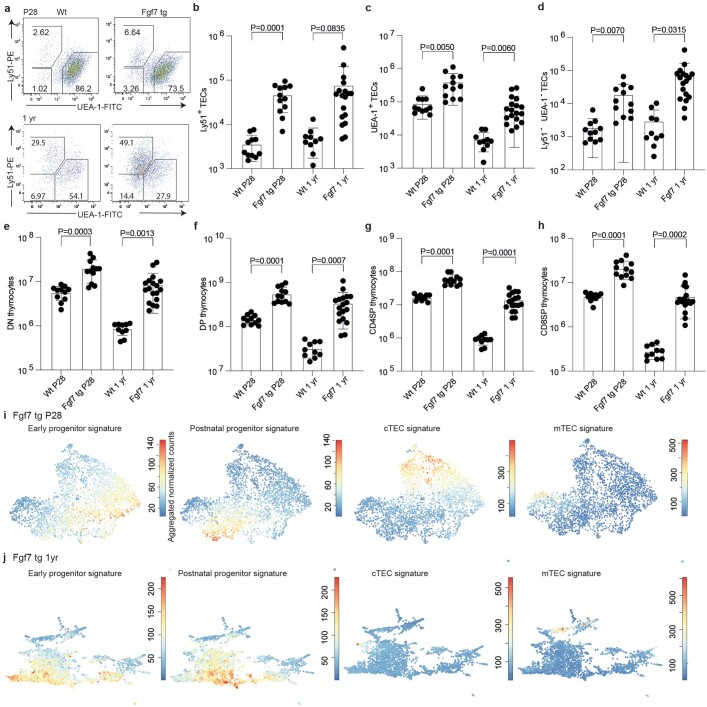
Changes in the thymic microenvironment upon autocrine Fgf stimulation. **a**, Representative flow cytometric profiles of Epcam^+^CD45^–^ TECs from wild-type (left panel) and *Foxn1:Fgf7* transgenic (right panel) mice at either 4-weeks (P28) or 1-year (1 yr) of age (top and bottom rows respectively); the percentages of individual TEC subpopulations are indicated in the respective gates. **b–d**, Numerical analysis of TEC subpopulations based on flow cytometry. For b-d, Wt P28, n = 11; Fgf7 tg P28, n = 12; Wt 1 yr, n = 10; Fgf7 tg 1 yr, n = 18. Data are shown as mean±s.e.m. **e–h**, Flow cytometric analyses of CD45^+^ thymocyte populations; DN, CD4^–^CD8^–^; DP, CD4^+^CD8^+^; CD4SP, CD4^+^CD8^–^; CD8SP, CD4^–^CD8^+^. For e-h, Wt P28, n = 11; Fgf7 tg P28, n = 12; Wt 1 yr, n = 10; Fgf7 tg 1 yr, n = 18. Data are shown as mean±s.e.m. t-test; two-sided; P values are indicated. **i, j**, UMAP representation of progenitor and mature TEC populations in *Foxn1:Fgf7* transgenic mice at (**i**) P28 and (**j**) 1 year of age. Source data

**Extended Data Fig. 13:**
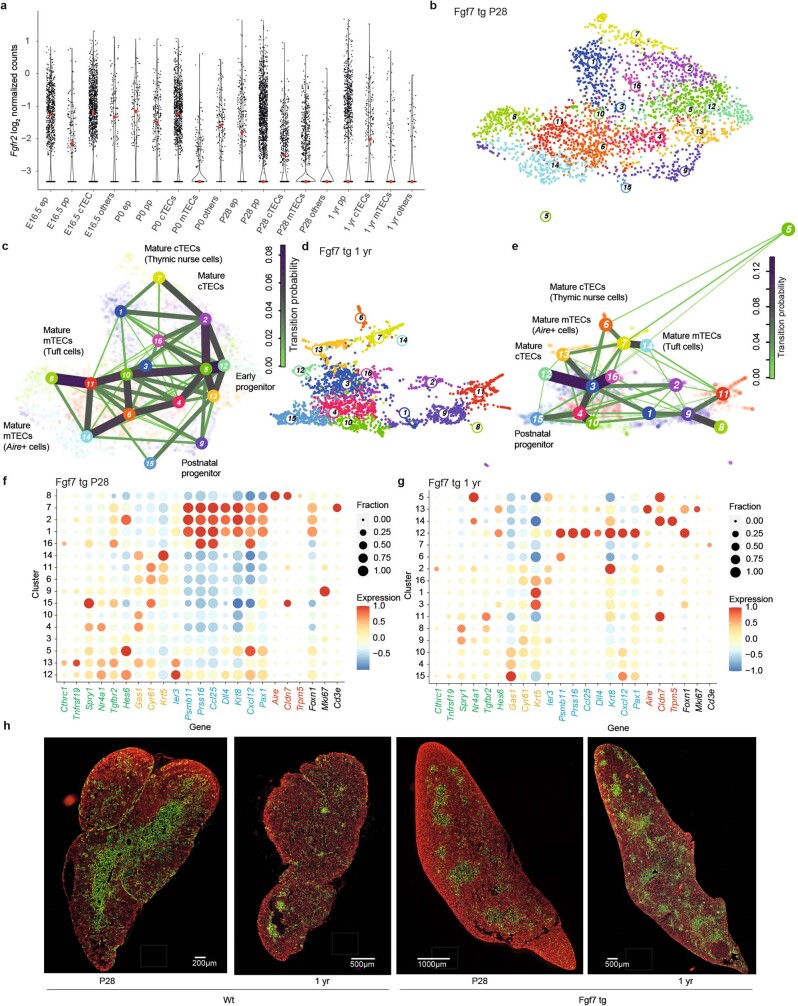
Effect of continuous Fgf7 signalling on the TEC compartment in young and old mice. **a**, Expression of the *Fgfr2* gene in cells of different TEC subsets of mice at different time points (E16.5 EP, n = 721 cells; E16.5 PP, n = 166 cells; E16.5 cTECs, n = 1,159 cells; E16.5 others, n = 141 cells; P0 EP, n = 136 cells; P0 PP, n = 226 cells; P0 cTECs, n = 924 cells; P0 mTECs, n = 311 cells; P0 others, n = 148 cells; P28 EP, n = 292 cells; P28 PP, n = 2,302 cells; P28 cTECs, n = 554 cells; P28 mTECs, n = 2,338 cells; P28 others, n = 303 cells; 1yr PP, n = 981 cells; 1yr cTECs, n = 225 cells; 1yr mTECs, n = 209 cells; 1 yr others, n = 87 cells). Data are presented as violin plots; the red dots indicate median expression levels. Negative cells are given a pseudo-count of 0.1. scRNA-seq datasets of barcoding mice across different time points were merged and normalized by downscaling to 1,500 transcript counts in order to calculate the log_2_-normalized transcript counts for *Fgfr2*. **b**, **d**, UMAP representation of transcriptome similarities between individual TECs isolated from thymi of P28 (**b**), or 1 year-old (**d**) *Foxn1:Fgf7* transgenic mice. **c**, **e**, Cluster designations deduced by VarID indicating the transcriptional relationships in terms of VarID-derived transition probabilities; connections with probabilities P > 0.001 are shown and the transition probabilities are indicated by line thickness and colouring. For orientation purposes, the major cell populations are also indicated. In panel (e), cluster 5 represents cells derived from ectopic parathyroid tissue. **f**, **g**, Expression profiles of TEC clusters for the indicated signature genes and the two time points. The fractions of each cluster expressing a particular gene and their respective expression levels are depicted according to the scales shown on the right. Dot colour represents the z-score of the mean expression levels of the gene in the respective cluster and dot size represents the fraction of cells in the cluster expressing the gene; gene names are coloured according to shared expression patterns (EP: green; PP: orange; cTEC, blue; mTEC, red; other genes of interest, black). z-scores above 1 and below -1 are replaced by 1 and -1 respectively. **h**, Immunohistochemical analysis of thymic lobes of wild-type (wt) and *Foxn1:Fgf7* transgenic mice at two different time points. Sections were stained with anti-Keratin 5 (green) and anti-Keratin 18 (red) antibodies, marking medullary and cortical compartments. Scale bars are indicated; panels are representative of 4 mice.

Next, we combined scRNA-seq and lineage analysis to examine the cellular composition of the TEC compartment under conditions of continuous fibroblast growth factor (Fgf) signalling, which is known to regulate the size of the TEC compartment. For instance, whereas functionally mature TECs are generated in the absence of *Fgfr2b*, the overall size of the TEC compartment is small, resulting in a hypoplastic thymus^53^; by contrast, mice treated with pharmacological doses of the Fgfr2b ligand KGF, the human homologue of Fgf7, exhibit an increase in the number of TECs^54,55^. However, it is not known whether Fgf stimulation targets progenitors, mature TECs or both. To examine this question, we generated several mouse models for continuous autocrine provision of an Fgfr2b ligand in the thymus. We established that, under physiological conditions, the extent of Fgf signalling in TECs (Extended Data Fig. 9) is determined by limiting levels of ligand(s), rather than the receptor (Extended Data Fig. 10); notably, we found that pharmacological supplementation of the Fgfr2b ligand Fgf7 could be mimicked in vivo by ectopic expression of Fgf7 in the TECs of *Foxn1-Fgf7* transgenic mice (Extended Data Fig. 11). Continuous autocrine provision of Fgf7 within the epithelial compartment in this transgenic model increased the number of TECs and thymocytes (Fig. 3a–c) and resulted in a massive and sustained increase in thymus size (Fig. 3d). Except for an increase in the number of Ly51^+^ TECs at P28 (Extended Data Fig. 12a–d) and a small reduction in the CD4/CD8 double-positive thymocyte compartment in old age (Extended Data Fig. 12e–h), thymopoiesis occurred normally in transgenic mice. Age-related diminution of thymopoiesis still occurred under conditions of chronic Fgf stimulation; however, the thymus of aged transgenic mice remained much larger than that of P28 wild-type mice (Fig. 3d). It appears therefore that reduced provision of Fgfr2b ligands contributes to the age-related progressive diminution of TEC numbers. Of note, expression levels of the *Fgfr2* genes in the different TEC populations were highest in early progenitors and cTECs at all time points (Extended Data Fig. 13a).

At the P28 and 1-year time points, the Fgf-stimulated thymic microenvironment also exhibited considerable heterogeneity, in terms of both transcriptional diversity and cluster size (Extended Data Fig. 13b–g). As indicated by the changes in flow cytometry profiles (Extended Data Fig. 12a), the proportion of cells exhibiting the cTEC signature was increased at P28, as was the proportion of early progenitors (Extended Data Fig. 12i). In aged transgenic mice, the TEC compartment exhibited the indistinct phenotype of aged postnatal progenitors (Extended Data Fig. 12j) that was observed in their wild-type siblings (Fig. 1j). The relative shifts in populations under conditions of continuous Fgf stimulation away from mTECs at P28 and away from mature cell types at 1 year of age (Extended Data Fig. 12j) were also apparent from the representation of cell clusters in the coordinate system discriminating the transcriptional signatures of progenitor cells and mature TECs (Fig. 3e–g), a feature summarized in Fig. 3h.

The lineage relationships of Fgf-stimulated TECs at P28 (Fig. 3i) showed the presence of barcodes that were shared by both types of progenitors and mature cTECs and mTECs (for example, barcode 43) and of barcodes that connected both progenitor types and cTECs (for example, barcode 78). The lineage structure in 1-year-old mice (Fig. 3j) showed a large number of progenitors that did not give rise to differentiated progeny (barcode 119). Other postnatal progenitors gave rise to either cTECs (barcode 116) or mTECs (barcode 8). In contrast to the dominance of lineage-biased progenitors, bipotent progenitors were rare (barcode 113). Collectively, our data indicate that TEC progenitors are not depleted by autocrine Fgf stimulation and actively contribute to the microenvironment in aged mice.

## Conclusions

Our study provides firm evidence for the contribution of early and postnatal bipotent progenitors to the formation and maintenance of the thymic epithelial microenvironment and reveals several new aspects of TEC biology. First, the two progenitor populations, an early cTEC-biased progenitor type and a postnatal mTEC-biased progenitor type, are already born during embryonic development and co-exist at E16.5, suggesting that some bipotent postnatal progenitors may be descendants of the early progenitor population (Fig. 3k). Second, the identification of private barcodes in both progenitor populations suggests that not all TEC progenitors are active at the same time, a phenomenon that is referred to as dormancy and known from other stem cell systems^56^. Third, the presence of private barcodes in mature cTECs and mTECs suggests the possibility that their corresponding progenitor(s) have been lost, in line with the notion that not all progenitor cells are long-lived^24,25^. Our current barcoding scheme does not allow us to determine whether these mature TECs can self-renew; resolution of this question awaits the use of an inducible version of the current marking scheme. Fourth, because the half-life of TECs is measured in weeks^7^, we conclude that long-term maintenance of the TEC compartment is associated with the activity of the postnatal progenitors identified here. Fifth, although continuous stimulation of thymic epithelia via autocrine secretion of an Fgfr2b ligand greatly increases the number of TECs, the qualitative characteristics of the thymic epithelium remain the same. However, the identification of progenitor populations provides new opportunities for focused pharmacological interventions to modulate the activity of the thymic microenvironment.

## Methods

### Mice

C57BL/6 mice were maintained in the Max Planck Institute of Immunobiology and Epigenetics. *Foxn1-eGFP*^57^, *Foxn1-cre*^58^, *Rosa26-LSL-EYFP*^59^, *Foxn1-s-Fgfr2IIIb*^60^, *pLck-cre*^61^, *Rosa26-LSL-Cas9-EYFP*^62^ and *Foxn1-mCardinal*^9^ transgenic mice have been described previously. The *Foxn1-Fgf7* transgene was created by inserting a cDNA fragment corresponding to nucleotides 347–934 in GenBank accession number NM_008008 as a NotI fragment into pAHB14 (ref. ^63^); in some aged female *Foxn1-Fgf7* transgenic mice (FVB/N-tg(Foxn1-Fgf7)1^Tbo^/Mpie), the two thymic lobes were asymmetric in size and shape; these mice were not included in our analysis. The *Foxn1-Fgfr2IIIb* transgene was created by inserting a cDNA fragment corresponding to nucleotides 1214–3366 in GenBank accession number NM_201601.2 as a NotI fragment into pAHB14 (ref. ^63^) and used to generate transgenic mice (FVB/N-tg(Foxn1-Fgfr2)1^Tbo^/Mpie). The *hU6-sgRNA*^*Hprt*^ transgene was cloned as a NotI fragment into the Bluescript vector and consists of the human *U6* promotor (nucleotides 1–264 in GenBank accession number JN255693) followed by the mouse *Hprt* target sequence (5′-GATGGGAGGCCATCACATTGG-3′; nucleotides 255–274 in GenBank accession number J00423), the sgRNA backbone (nucleotides 218–139 (reverse complement) in Addgene plasmid 42250) and a short 3′ sequence (TTTTTTGGAA); for injection into fertilized eggs, the construct was linearized with SacI. Transgenic mice were generated on an FVB/N background (FVB/N-tg(hU6-sgRNA-Hprt)1^Tbo^/Mpie) and subsequently backcrossed to a C57BL/6J background. For timed matings, the day of plug detection was designated as E0.5. Genotyping information is summarized in Supplementary Table 15. Mice were kept in the animal facility of the Max Planck Institute of Immunobiology and Epigenetics under specific pathogen-free conditions. All animal experiments were performed in accordance with the relevant guidelines and regulations, approved by the review committee of the Max Planck Institute of Immunobiology and Epigenetics and Regierungspräsidium Freiburg, Germany (licences 35-9185.81/G-12/85; 35-9185.81/G-16/67).

### KGF treatment

At the age of 4 weeks, male mice received nine intraperitoneal injections of KGF (Kepivance, Biovitrum, lot D120961G; 5 mg kg^–1^ body mass) at days 1, 2, 3, 8, 9, 10, 15, 16 and 17; the mice were killed on day 21.

### Histology

Embryos for RNA in situ hybridization (ISH) were fixed in 4% paraformaldehyde (PFA) and subsequently embedded in paraffin using standard techniques.

### qPCR

The Applied Biosystems 7500 Fast system was used to detect the signal generated with gene-specific primers combined with 5′-FAM (6-carboxyfluorescein)-labelled hydrolysis probes from Universal Probe Library (Roche). Primer sequences were as follows: *Fgf7*, 5′-TGGCTGACACCATGACTAGC-3′ and 5′-GGCTACAGGCTGTCGTTTTT-3′ (probe 42); *Fgf10*, 5′-CGGGACCAAGAATGAAGACT-3′ and 5′-GCAACAACTCCGATTTCCAC-3′ (probe 80); *CD31* (*Pecam*), 5′-CGGTGTTCAGCGAGATCC-3′ and 5′-ACTCGACAGGATGGAAATCAC-3′ (probe 45); *Ly51* (*Enpep*), 5′-TGGACTCCAAAGCTGATCCT-3′ and 5′-TCAGCCCATCTGACTGGAAT-3′ (probe 83). Expression levels were normalized to those of *Hprt*, using primers 5′-TCCTCCTCAGACCGCTTTT-3′ and 5′-CCTGGTTCATCATCGCTAATC-3′ (probe 95).

### RNA ISH

RNA ISH on paraffin sections was performed using DIG-labelled probes as described^64^. Sequence coordinates in GenBank accession numbers were as follows: *Foxn1*, nucleotides 2181–3584 in XM_006532266.3; *Fgf7*, nucleotides 153–877 in NM_008008.3; *Fgf10*, nucleotides 859–1570 in NM_008002.3; *Fgfr1*, nucleotides 761–1614 in NM_001079909.2; *Fgfr2*, nucleotides 328–800 in EF143340; *Fgfr2_exon3b*, nucleotides 1819–1964 in NM_201601.2; *Fgfr2_exon3c*, nucleotides 2169–2306 in NM_010207.2; *Hspb1*, nucleotides 224–632 in NM_013560.2; *Trpm5*, nucleotides 499–962 in NM_020277.2.

### Immunohistochemistry

Thymi were fixed in 4% PFA, washed in PBS, incubated in 20% sucrose overnight and embedded in OCT. Sections of 8–10 µm were dried overnight at room temperature and before staining were moistened in PBS followed by a 30-min blocking step (PBS with 0.5% BSA, 0.2% Tween and anti-mouse IgG (1:50)). Antibody staining was performed at room temperature in staining buffer (PBS with 0.5% BSA, 0.2% Tween and 3% serum). Sections were stained for 2 h with primary antibodies (Supplementary Table 6) and then for 45 min with secondary antibodies and streptavidin. Sections were washed with PBS between incubations. After staining, sections were mounted in Fluoromount G.

### Image analysis

Images were acquired on Zeiss microscopes (Axioplan 2 or Imager Z1 with ApoTome attachment) equipped with AxioCam MRc 5 cameras.

### Flow cytometry

To generate single-cell suspensions for analytical and preparative flow cytometry of TECs, the procedures described in refs. ^18,65^ were followed. Relevant staining reagents are listed in Supplementary Table 16. The enzymatic cocktail required to liberate TECs destroys the extracellular domains of the CD4 and CD8 surface markers (but not that of the CD45 molecule); hence, when analysis of thymocyte subsets was desired, thymocyte suspensions were prepared in parallel by mechanical liberation, achieved by gently pressing thymic lobes through 40-µm sieves. To isolate thymic mesenchymal and endothelial cells, the cell suspension of total thymocytes was depleted of CD45^+^ cells; the EpCAM^–^CD45^–^ cell population was stained for Ly51 and CD31 to purify EpCAM^–^CD31^–^Ly51^+^ mesenchymal and EpCAM^–^CD31^+^Ly51^–^ endothelial cells. Cell sorting and analytical flow cytometry were carried out using MoFlow and Fortessa instruments, respectively (both from Dako Cytomation-Beckman Coulter); flow cytometry experiments were carried out using FACSDiva and FlowJo software. The fraction of *Foxn1*-expressing cells was determined by eGFP fluorescence emanating from the *Foxn1-eGFP* transgene^57^, which faithfully recapitulates acute levels of *Foxn1* expression^18,34^. The thymopoietic index was calculated by dividing the total number of thymocytes by the number of TECs.

### Single-cell RNA amplification and library preparation

scRNA-seq was performed using the CEL-Seq2 method^45^ with several modifications^44^. A fivefold volume reduction was achieved using a nanolitre-scale pipetting robot (Mosquito HTS, TTP Labtech)^66^. EpCAM^+^CD45^–^ TECs were sorted into 384-well plates containing 240 nl of primer mix^67^ and 1.2 μl of mineral oil (Sigma-Aldrich) replacing Vapor-lock. Sorted plates were centrifuged at 2,200*g* for 3 min at 4 °C, snap-frozen in liquid nitrogen and stored at −80 °C before further processing. To convert RNA into cDNA, 160 nl of reverse-transcription reaction mix including 0.4 µM template-switch oligonucleotide (5′-AAGCAGTGGTATCAACGCAGAGTGAATrGrGrG-3′; adapted from ref. ^68^) and 2.2 μl of second-strand reaction mix were added.

For the purpose of simultaneous transcriptome and barcode analysis, the volume of each well was equally split in half; that is, 1.1 µl per well was transferred to a new 384-well plate. The original plate (including the mineral oil) was used for barcode analysis, while the copy of the plate was used for analysis of individual cell transcriptomes.

For barcode analysis, all transcripts in each well were amplified by template-switch PCR (5′-AAGCAGTGGTATCAACGCAGAGT-3′ (template-switch oligonucleotide primer) and 5′-CAGAGTTCTACAGTCCGA-3′ (short P5 primer)), followed by amplification of the scar region of *Hprt* transcripts (5′-GCCGGTAATACGACTCACTATAGGGAGTTCTACAGTCCGACGATC-6bp UMI-6bp cell barcode-CCAGTTAAAGTTGAGAGATCATCTCC-3′ (*Hprt* barcoding primer), 5′-GCCTTGGCACCCGAGAATTCCATAGCGATGATGAACCAGGTTATGACC-3′ (*Hprt* short P7 primer)) using the PrimeSTAR GXL system (Takara Bio). Libraries were completed by addition of full-length adaptors by PCR (RP1, RPI1-48 TruSeq Small RNA Illumina adaptor sequences). Libraries from 96 cells were pooled before clean-up. In total, 32 libraries (E16.5 time point), 24 libraries (P0 time point), 28, 40 and 80 libraries (three P28 time points), 32 libraries (1-year time point), 44 libraries (*Foxn1-Fgf7* transgenic mice, P28 time point) and 60 libraries (*Foxn1-Fgf7* transgenic mice, 1-year time point) were sequenced on the MiSeq sequencing system (2 × 300 bp) at a depth of ≥4,000 reads per cell.

Transcriptomes were generated as described previously^66^; for the P28 wild-type dataset from a non-barcoded mouse, the whole sample volume was used for transcriptome generation. cDNAs from 96 cells were pooled before clean-up and in vitro transcription, generating four libraries from one 384-well plate. For all purification steps, 0.8 μl of AMPure/RNAClean XP beads (Beckman Coulter) was used per 1 μl of sample, including in library clean-up.

In total, 32 libraries (E16.5 time point), 24 libraries (P0 time point), 28, 40 and 80 libraries (three P28 time points), 28 libraries (non-barcoded dataset of P28 time point), 32 libraries (1-year time point), 44 libraries (*Foxn1-Fgf7* transgenic mice at P28 time point) and 60 libraries (*Foxn1-Fgf7* transgenic mice at 1-year time point) (each library was generated by pooling 96 cells) were sequenced on the Illumina HiSeq 2500 or NovaSeq 6000 sequencing system (paired-end multiplexing run, high-output mode) at a depth of ~170,000 reads per cell.

### Quantification of transcript abundance

Paired-end reads were aligned to the transcriptome using bwa (version 0.6.2-r126) with default parameters^69^. The transcriptome contained all gene models based on the mouse ENCODE VM9 release downloaded from the UCSC genome browser, comprising 57,207 isoforms, with 57,114 isoforms mapping to fully annotated chromosomes (1 to 19, X, Y, mitochondria). All isoforms of the same gene were merged to a single gene locus. Furthermore, gene loci overlapping by >75% were merged to larger gene groups. This procedure resulted in 34,111 gene groups. The right mate of each read pair was mapped to the ensemble of all gene loci and to the set of 92 ERCC spike-ins in the sense direction^70^. Reads mapping to multiple loci were discarded. The left read contained the barcode information: the first six bases corresponded to the cell-specific barcode followed by six bases representing the unique molecular identifier (UMI). The remainder of the left read contained a poly(T) stretch. The left read was not used for quantification. For each cell barcode, the number of UMIs per transcript was counted and aggregated across all transcripts derived from the same gene locus. On the basis of binomial statistics, the number of observed UMIs was converted into transcript counts^71^.

### scRNA-seq data analysis

Clustering analysis and visualization of all datasets were performed with the VarID algorithm^46^. Cells with a total number of transcripts of <1,000 (1-year wild-type dataset), <1,500 (wild-type and *Foxn1-Fgf7* transgenic P28 datasets) and <3,000 (wild-type E16.5, wild-type P0 and *Foxn1-Fgf7* transgenic 1-year datasets) were discarded, and the count data of the remaining cells were normalized by downscaling. Notably, before normalization, cells yielding transcriptomes containing >2% *Kcnq1ot1* transcripts, a previously identified marker of low-quality cells, were removed from the analysis^72^. Moreover, transcripts correlating to *Kcnq1ot1* with a Pearson’s correlation coefficient of >0.65 were also removed. Furthermore, mitochondrial genes as well as *Hprt* were excluded. The following genes and correlating gene groups were removed from the analysis using the CGenes parameter (all datasets): *Jun*, *Fos* and predicted genes with *Gm* identifiers. For the wild-type P28 datasets, *Malat1*, *Xist* and *Neat1* were excluded using the FGenes parameter. A pruned *k* nearest neighbour (kNN) matrix was inferred using the pruneKnn function of VarID with default parameters except alpha (set to 1) and no_cores (set to 10). The UMAP representation was used for cell cluster visualization^73^. Transition probabilities between clusters were computed using the transitionProbs function of VarID with the *P* value set to 0.001. Differentially expressed genes between two subgroups of cells were identified similarly to in a previously published method^74^. First, negative binomial distributions reflecting the gene expression variability within each subgroup were inferred on the basis of the background model for the expected transcript count variabilitycomputed by the RaceID3 algorithm^44^. Using these distributions, a *P* value for the observed difference in transcript counts between the two subgroups was calculated and corrected for multiple testing with the Benjamini–Hochberg method.

### High-resolution lineage tracing

The lineage tracing method developed here was based on CRISPR–Cas9-mediated scarring in exon 3 of the *Hprt* gene (Fig. 2a). Repair of Cas9-induced double-strand breaks results in a number of different sequence outcomes, which we refer to here as barcodes because they indelibly mark all cell progeny. Most barcodes carry deletions (Fig. 2b), preventing secondary modifications of their sequence; however, in rare instances, we recorded cells with two barcodes of similar sequence, presumably originating from ongoing modifications of the target sequence or from relaxed X-chromosome inactivation. In bulk and single-cell analyses of male mice, barcodes can be unambiguously read out on both the DNA and transcript level from the single X chromosome. In female mice, which carry two X chromosomes, DNA analysis at the level of cell populations yields ambiguous results; however, for single-cell analysis, the phenomenon of dosage compensation enables unambiguous barcode analysis at the RNA level. For the bulk analyses described here, we therefore used only male mice, whereas single-cell transcriptome analyses were conducted in both male and female mice. In the *hU6-sgRNA*^*Hprt*^; *Foxn1-cre*; *Rosa26-LSL-Cas9-EYFP* triple-transgenic mice used here, TECs are marked in early embryogenesis, as soon as *Foxn1* expression begins; our previous analysis indicated that, at the onset of *Foxn1* expression, the thymic rudiment harbours ~4,000 epithelial cells^9^, thus placing an upper limit on the number of barcodes that can be observed in TECs at later developmental stages. However, the observed number of barcodes was three- to fourfold lower, presumably because the outcome of the repair process is not random; hence, some barcodes, although independently generated, are identical in sequence. The different frequencies of barcode generation must be taken into account when reconstructing lineage relationships; rare barcode sequences are more informative than frequently generated barcodes. On the basis of previous experiments using our *Foxn1-cre* transgenic line and the accessibility of the *Rosa26* locus in TECs^34^, we assume that the overwhelming majority of TECs (>95%) are barcoded early in embryogenesis. However, we cannot exclude the possibility that some cells in the thymic rudiment are recruited into the *Foxn1*-positive lineage at later stages of development. If so, this would however only lessen the ability to identify clonal relationships across all time points.

### Single-cell barcoding analysis

Paired-end fastq files were used for the identification of scar sequences in single cells. The first six bases of the left read contained the UMI information, followed by six bases representing the cell barcode. The remainder of the left read contained the *Hprt* scar sequence. The right mate of the paired-end reads also contained the overlapping *Hprt* scar sequence; that is, both reads contained the full scar sequences. Primers (forward, 5′-GCTCGAGATGTCATGAAGG-3′; reverse, 5′-GGGGGGCTATAAGTTCTT-3′) were used to extract the targeted region of the *Hprt* gene containing the edited sequence. Because both the left and right mates of the paired-end reads contain the full scar sequences, only sequences that appeared in both paired-end reads were used for further analysis. Moreover, only cells yielding ≥200 reads were included in the analysis; cells were excluded from further analysis if more than one scar sequence was detected in male cells (the threshold for the second sequence was set at ≥10% of the major sequence) and if more than two scar sequences were detected in female cells (the threshold for the third sequence was set at ≥10% of the second sequence).

### Barcodes shared by cTECs and mTECs

DNA was isolated from sorted EpCAM^+^CD45^–^Ly51^+^UEA-1^–^ (cTEC) and EpCAM^+^CD45^–^Ly51^–^UEA-1^+^ (mTEC) populations from each mouse, and the region of exon 3 of the *Hprt* gene was amplified using the following primers: 5′-ACACTCTTTCCCTACACGACGCTCTTCCGATCTTTCATAGAGACAAGGAATGTGTCC-3′ (forward, P5-DD302)and 5′-GTGACTGGAGTTCAGACGTGTGCTCTTCCGATCTAGTTGATTATGTAGCAtAGTTTGACAAG-3′ (reverse, P7-DD305). Libraries were sequenced at a depth of ~250,000 reads per sample on the MiSeq sequencing system (2 × 300 bp).

Next, a table containing the counts of all barcodes across cTECs and mTECs for each mouse (*n* = 33 mice) was constructed, and the frequency distribution of barcodes was determined (Fig. 2d). To quantify enrichment of shared barcodes between the cTEC and mTEC samples for a given mouse, we first extracted the set of all barcodes *B*_*i*_ for sample *i* that were observed no more than twice in all animal samples; then, we determined the number of barcodes within set *B*_*i*_ co-occurring in another sample *j* and divided this by the number of barcodes in *B*_*i*_ to compute the co-occurrence probability of rare barcodes, termed *P*_*ij*_:

$${P}_{{ij}}=\frac{\sum _{k\in {B}_{i}}{\chi }_{{B}_{j}}\left(k\right)}{\sum _{k\in {B}_{i}}{\chi }_{{B}_{i}}\left(k\right)}$$

Here *χ*_*T*_(*x*) denotes the indicator function; that is, *χ*_*T*_(*x*) = 1 if *x* ∊ *Tχ*_*T*_(*x*) = 0 otherwise.

If *i* and *j* denote mTEC and cTEC samples from the same mouse, we would like to test whether *P*_*ij*_ is relatively increased compared with cases where *i* and *j* denote samples from different mice.

An increased co-occurrence probability in corresponding samples compared with all other mice is indicative of a common barcode repertoire and is interpreted to mean that cTEC and mTEC populations in a mouse arise from common progenitors marked by particular barcodes; conversely, a similar ratio across all animals suggests the random occurrence of rare barcodes and argues against a common origin. The observation that some of the rare barcodes are not shared by cTEC and mTEC populations from the same mouse can be explained by the low frequency of these rare barcodes, resulting in sampling dropouts, but may also have a biological explanation, for example, if either mTEC or cTEC progeny derived from the same progenitor have died out. In this context, it is worth noting that, without prefiltering based on barcode frequency, the fraction of barcodes shared by mTEC and cTEC populations was >50%.

To quantify a single enrichment value *E*_*m*_ for a given animal *m*, the ratios calculated for a corresponding pair of cTEC and mTEC samples, *m*_cTEC_ and *m*_mTEC_, from the same mouse were divided (with cTECs or mTECs as a reference) by the average of the ratios for pairings involving either the respective cTEC or mTEC sample and a sample from any other animal, excluding the samples from animal *m*. The enrichment value *E*_*m*_ is then calculated as the maximum of these ratios with mTECs or cTECs from the same animal as a reference:

$$\begin{array}{ccc}{E}_{m} & = & {\rm{\max }}(\frac{{P}_{{m}_{{\rm{cTEC}}}{m}_{{\rm{mTEC}}}}}{\frac{1}{N-2}\sum _{j\ne {m}_{{\rm{cTEC}}}{\vee j\ne m}_{{\rm{mTEC}}}}{P}_{{m}_{{\rm{cTEC}}}j}},\\  &  & \frac{{P}_{{m}_{{\rm{mTEC}}}{m}_{{\rm{cTEC}}}}}{\frac{1}{N-2}\sum _{j\ne {m}_{{\rm{cTEC}}}{\vee j\ne m}_{{\rm{mTEC}}}}{P}_{{m}_{{\rm{mTEC}}}j}})\end{array}$$

Here *N* is the total number of samples and ∧ indicates logical conjunction. In the summation of the denominators, we exclude pairings involving the mTEC or cTEC samples for animal *m*.

The enrichment values of shared rare barcodes for each mouse were then plotted for each individual mouse grouped by age bin in Fig. 2f. Note that the analysis of bulk populations is most robust with respect to sampling when the numbers of cTECs and mTECs are approximately equal, as is the case for the P14 time point^9^. In situations where only a small number of barcodes are recovered, a diminished degree of co-occurrence is probably the result of sampling dropouts and the associated reduced statistical power.

### Lineage analysis

To quantify the enrichment of shared barcodes between different populations within the TEC compartment at single-cell resolution, we first identified the cell clusters representing early progenitors, postnatal progenitors, and mature cTECs and mTECs in the scRNA-seq analysis of the individual mice. For a given mouse, we determined the barcode repertoire and counted the number of cells for each barcode; in the rare instances where a pair of barcodes was observed, we used the combination of barcodes for quantification. Cells with more than two barcodes were discarded, as this situation is the result of cell doublets and/or sequencing errors. For each barcode (or pair of quantified barcodes), we next determined its frequency for each TEC population in a given sample. These frequencies were compared with background barcode frequencies derived from the barcode distribution quantified from the bulk DNA sequencing data (*n* = 33; Fig. 2d), averaging across all samples. We considered sampling dropouts as a reference background model for technical variability and, therefore, assessed significant over-representation of barcodes on the basis of the estimated probability mass of a binomial distribution with a probability parameter informed by the barcode frequency derived from the bulk sequencing experiments. It is well known that UMI-based abundance derived from scRNA-seq data can be modelled by a negative binomial distribution without explicitly modelling zero inflation and that variability in genes (or barcodes) with low expression is described well by the binomial noise component^71^.

If *n*_*b*,*i*_ is the number of times barcode *b* was observed in bulk DNA sequencing sample *i*, the background frequency of barcode *f*_*b*_ was calculated as

$${f}_{b}=\frac{1}{N}\sum _{i\in S}\frac{{n}_{b,i}}{\sum _{k\in B}{n}_{k,i}}$$

Here *B* denotes the set of all barcodes, *S* denotes the set of all samples and *N* is the size of *S*.

Assuming binomial sampling statistics, we then calculated the *P* value *P*_*b*,*i*_ for the observed number of cells *c*_*b*,*i*_ with barcode *b* among all barcode-carrying cells in sample *i* obtained by scRNA-seq as the right tail probability of the binomial distribution with background probability *f*_*b*_ and the total number of barcode-carrying cells as parameters:

$${P}_{b,i}=\mathop{\sum }\limits_{j={c}_{b,i}}^{{C}_{i}}B\,(j|\,{f}_{b}\,,\,{C}_{i}\,{\rm{w}}{\rm{i}}{\rm{t}}{\rm{h}}\,{C}_{i})=\sum _{k\in B}{c}_{k,i}$$

These *P* values were further corrected for multiple testing by the Benjamini–Hochberg method, considering all *P* values for all detected barcodes in a given dataset. For cells with two barcodes, we calculated *P* values accordingly after multiplying the background frequencies of the co-occurring barcodes. Barcodes were considered informative if their *P* value indicated a significant deviation from the expected frequency (*P*_*b*,*i*_ < 0.001).

### Gene set analysis

Population-specific gene sets were derived by performing differential gene expression analysis of clusters representing early progenitors (c5), postnatal progenitors (c1 and c6), cTECs (c4) and mTECs (c12, c2, c7, c9, c10 and c18) versus all other clusters from data for 4-week-old (P28) mice using the diffexpnb function of the RaceID3 package^44^. Genes with adjusted *P* < 0.01 and log_2_(fold change) > 1 were included in gene sets, from which genes with *Gm* and *Rik* identifiers were excluded. Overlapping genes between early progenitor and cTEC gene sets were excluded from the early progenitor gene set. Genes included in the gene sets of each of the four populations are listed in Supplementary Tables 1–4.

Although the transcriptional profiles of the progenitor clusters differed from those of the mature TEC subsets by the expression of heat shock protein genes, these genes were not included in the final lists, as they did not distinguish between the early and postnatal progenitors. The final gene sets were analysed for enriched biological processes using the Database for Annotation, Visualization and Integrated Discovery (DAVID) version 6.8 Analysis Wizard annotation tool^75,76^. In the representation of gene set ratios, clusters expressing T cell progenitor-related genes (representing thymic nurse cells) and parathyroid-associated genes (representing ectopic parathyroid tissue) were excluded.

### scRNA-seq data comparisons

The present data were compared with publicly available scRNA-seq data for TECs isolated from mice of different ages^1^. To do this, the raw count matrices and metadata describing the nine subtypes of TECs were obtained through the Bioconductor data package MouseThymusAgeing (https://bioconductor.org; 10.18129/B9.bioc.MouseThymusAgeing). Data normalization, dimensionality reduction and visualization with UMAP were then performed using the default parameters of the scRNA-seq data analysis CRAN package Seurat version 3 (ref. ^77^).

### Statistical analysis and reproducibility

Two-tailed *t* tests were used to determine the significance levels of differences between the means of two independent samples, considering equal or unequal variance as determined by the *F* test. For multiple tests, the conservative Bonferroni correction was applied. For all analyses, several biological replicas were studied; numbers of replicas are indicated in the figures and/or figure legends. No statistical methods were used to predetermine sample sizes; blinding and randomization were not used. 

### Reporting summary

Further information on research design is available in the Nature Research Reporting Summary linked to this paper.

## Online content

Any methods, additional references, Nature Research reporting summaries, source data, extended data, supplementary information, acknowledgements, peer review information; details of author contributions and competing interests; and statements of data and code availability are available at 10.1038/s41586-022-04752-8.

### Supplementary information

Supplementary Tables This file contains Supplementary Tables 1–16.

Reporting Summary

### Source data

Source Data Fig. 1

Source Data Fig. 2

Source Data Fig. 3

Source Data Extended Data Fig. 1

Source Data Extended Data Fig. 2

Source Data Extended Data Fig. 6

Source Data Extended Data Fig. 9

Source Data Extended Data Fig. 10

Source Data Extended Data Fig. 11

Source Data Extended Data Fig. 12

## Data Availability

The primary read files as well as expression count files for the scRNA-seq datasets reported in this paper are available to download from GEO (accession number GSE106856). Source data are provided with this paper.
