# Publisher Correction: Developmental dynamics of two bipotent thymic epithelial progenitor types

**DOI:** 10.1038/s41586-024-07129-1

**Published:** 2024-02-01

**Authors:** Anja Nusser, Sagar, Jeremy B. Swann, Brigitte Krauth, Dagmar Diekhoff, Lesly Calderon, Christiane Happe, Dominic Grün, Thomas Boehm

**Affiliations:** 1 Department of Developmental Immunology, Max Planck Institute of Immunobiology and Epigenetics, Freiburg, Germany; 2 Quantitative Single Cell Biology Group, Max Planck Institute of Immunobiology and Epigenetics, Freiburg, Germany; 3 Department of Medicine II, University Hospital Freiburg, Freiburg, Germany; 4 Würzburg Institute of Systems Immunology, Max Planck Research Group at the Julius-Maximilians-Universität Würzburg, Würzburg, Germany; 5 Helmholtz Institute for RNA-Based Infection Research (HIRI), Helmholtz Centre for Infection Research (HZI), Würzburg, Germany; 6 Faculty of Medicine, University of Freiburg, Freiburg, Germany; 7 Present Address: Institute of Molecular Pathology, Vienna, Austria

**Keywords:** Immunology, Cell biology

Correction to: *Nature* 10.1038/s41586-022-04752-8 Published online 25 May 2022

In the version of the article initially published, in Fig. 2g, the designations of the columns at the bottom of the figure were inverted. There were also errors in Fig. 2j and Extended Data Fig. 7g,h, where the designations for EP and PP were inadvertently transposed. Accordingly, in the “Combining scRNA-seq and barcode tracing” section, the sentence “At P28, cells with the transcriptional signature of early progenitors predominantly gave rise to mTECs (barcode 86) rather than both mTECs and cTECs (barcode 85); the number of postnatal progenitors biased towards mTEC differentiation (barcode 96) increased (Fig. 2j),…” has been amended to “At P28, cells with the transcriptional signature of early progenitors predominantly gave rise to mTECs (barcode 96) rather than both mTECs and cTECs; the number of postnatal progenitors biased towards mTEC differentiation (barcodes 85,86) increased (Fig. 2j),…”. In the Methods section, it was stated that “32 libraries” were sequenced for the P0 time point. It should have read “24 libraries”. These errors have been corrected in the HTML and PDF versions of the article. None of these errors affect the conclusions of the paper.

