# Supplementary material for: Developmental dynamics of two bipotent thymic epithelial progenitor types
Source: Nature. 2022 May 25;606(7912):165–71. doi: 10.1038/s41586-022-04752-8 (PMC9159946; doi:10.1038/s41586-022-04752-8)
Supplement: Supplementary file 1 — This file contains Supplementary Tables 1–16. [file 41586_2022_4752_MOESM1_ESM.pdf]

nature portfolio

https://doi.org/10.1038/s41586-022-04752-8

Supplementary information

Developmental dynamics of two bipotent thymic epithelial progenitor types

In the format provided by the authors and unedited

Supplementary Table 1 | Gene set list for early progenitor.

Ackr4	Fstl1	Ogn	Wscd1
Adamts10	Gas1	Pak3	Xdh
Agrn	Gbp2	Palld	Zfp36
Aldh2	Gnaq	Pdpn	
Aldh6a1	Gpm6b	Penk	
Amotl1	Gprasp1	Plxdc2	
Amotl2	Gstm2	Pmp22	
Antxr1	H2-DMa	Prelp	
Apoe	Hes6	Prrg3	
Ar	Hic1	Prss23	
Bcam	Hsd17b10	Ptprz1	
Bcl11a	Igfbp2	Ptrf	
Bcl2	Igfbp3	Pygb	
Bmp4	Igfbp5	Rbp1	
Btg2	Igfbp7	Rnase4	
Cbx6	Iigp1	Scn1a	
Ccdc80	Il33	Serpinf1	
Cdh11	Irgm1	Serpinh1	
Cldn8	Isl1	Shisa2	
Clec11a	Itm2c	Slc2a13	
Clstn1	Kazald1	Sord	
Col18a1	Lamb1	Sparc	
Cpne8	Laptm4a	Spon2	
Cthrc1	Limch1	Spry1	
Cyp1b1	Ltbp3	Tcn2	
Dcn	Maged1	Tgfbr2	
Ddr1	Megf6	Tgfbr3	
Dhrs3	Meis1	Thbd	
Dlk2	Mgll	Thbs1	
Dnajc13	Mgp	Timp2	
Dpp6	Myl9	Tinagl1	
Dpysl2	Mylk	Tnfrsf19	
Dsc3	Nbl1	Tns1	
Egr1	Nell2	Tns3	
Eid1	Nfia	Trim29	
Fam129a	Nfib	Trp63	
Fam19a5	Nfix	Tspan9	
Fkbp9	Nr2f1	Twsg1	
Fmod	Nr4a1	Txnip	
Fos	Nrtn	Unc119	
Fosb	Ntrk3	Vmac	
Frmd6	Oat	Wls	

Supplementary Table 2 | Gene set list for postnatal progenitor.

Acta2	Myl9
Apoe	Nrbp2
Ascl1	S1pr3
Boc	Slc4a11
C1s1	Sox4
C3	Stat2
Cald1	Sult5a1
Ccl11	Tagln
Ccl21a	Tgfbi
Clca3a1	Tpm2
Col6a1	Wfikkn2
Col6a2	
Cyr61	
Ddx60	
Dpysl3	
Dst	
Emp2	
Fam101b	
Flna	
Fst	
Fzd2	
Gas1	
Glul	
Gpx3	
Gsn	
Hpgd	
Htra1	
Id1	
Ifi27l2a	
Igfbp4	
Igfbp5	
Irf7	
Isg15	
Itga6	
Itgb4	
Krt14	
Krt5	
Krt7	
Lamb3	
Lars2	
Lifr	
Mgp	

Supplementary Table 3 | Gene set list for cTECs.

Abat	Cxcl12	Ier3	Pepd	Snhg11
Adhfe1	Cxx1a	Ifi27	Pfn2	Snx3
Agpat3	Cyp26b1	Impact	Pithd1	Spock2
AI314180	Dact2	Inmt	Pla2g16	Spryd7
AI413582	Dcxr	Irf1	Plekhb1	Srebf1
Akap12	Diras2	Isg20	Plgrkt	St3gal6
Akap2	Dll4	Itgb2	Pltp	Stard5
Alcam	Dock8	Kcnk2	Ppp1r27	Syngr1
Aldh1l1	Dsp	Kitl	Prdx5	Synm
Aldh4a1	Dst	Krt18	Prr32	Tbata
Ank3	Dtx4	Krt8	Prss16	Tbc1d2b
Arhgef10l	Dusp28	Lamp2	Psat1	Tcf7
Bag3	Dynll2	Lancl1	Psma1	Thy1
Bahd1	Dynlt3	Lat	Psma5	Trbc1
Bicd2	Endou	Lck	Psma7	Trpm2
Bnip3l	Enpep	Lpcat2	Psmb10	Tsc22d1
Bok	Etnk1	Lpl	Psmb11	Tulp3
Btbd11	Exoc7	Ly75	Psmb4	Ufsp1
Calcoco1	Fabp5	Man2a1	Psmb9	Vat1
Cbr2	Fam213b	Mast4	Psmc2	Wnt4
Ccdc34	Fam49b	Mfsd12	Pttg1ip	Zfyve21
Ccl25	Fasn	Mgat3	Rab11fip4	
Ccr9	Fgf14	Mmp2	Rag1	
Cd4	Fmo1	Mreg	Rassf2	
Cd83	Foxn1	Mrpl3	Rassf5	
Cd8a	Foxo3	Mtss1l	Rbms3	
Cd8b1	Fth1	Myo5c	Rgcc	
Cdh4	Fxyd2	Nav2	Rnpepl1	
Cdk20	Gabarapl1	Ndrg3	S100a1	
Cdr2l	Gas6	Ndufa11	S100a10	
Cds1	Gatsl3	Nefm	Satb1	
Cisd3	Gch1	Neurl2	Scarf2	
Clic5	Ggh	Nin	Scd2	
Clip4	Got1	Nqo1	Scnn1a	
Cnn3	Gtf2e2	Nsmaf	Setd7	
Copz2	Hadhb	Nxn	Sfxn1	
Cpt1a	Hdac7	Ociad2	Sgpl1	
Creb3l2	Hexb	Oma1	Slc22a7	
Crip3	Hey1	P4ha3	Slc25a4	
Cstb	Hgsnat	Pax1	Slc36a1	
Cth	Hist1h2bc	Pds5b	Slc46a2	
Ctsl	Hpgd	Pdxk	Snap91	

Supplementary Table 4 | Gene set list for mTECs.

AA467197	Ccl6	Ehd1	Igfbp6	Mxd1	Rap1gap	Sprr1a
Adap1	Ccl9	Elf3	Igsf8	Myl7	Rarres1	Srgn
Adgb	Ccr7	Eno2	Il12a	Myo15b	Rasd1	Sspo
Aebp2	Cd52	Ero1lb	Il13	N4bp2l1	Reg3g	Sst
Ahcyl2	Cd70	Espn	Il1rn	Ncf1	Resp18	Syt1
Aif1l	Cdh17	Etv3	Il23a	Ncf4	Rnasel	Syt7
Aire	Cdhr5	Fabp4	Il2rg	Neat1	Rnf128	Tac2
Alas1	Cdkn1a	Fabp6	Ing1	Nfkbia	Rogdi	Tbc1d4
Ank	Cdkn1c	Fabp9	Inpp5b	Nos2	Rptn	Tfap2b
Ankrd33b	Cdkn2b	Fam25c	Insm1	Nostrin	S100a14	Tff3
Anxa10	Cdx1	Fam89a	Itgb8	Nptx2	S100a4	Tjp3
Aoc1	Chil1	Fcer1g	Kcnk6	Nsmce1	S100a8	Tkt
Apoa1	Ckmt1	Fezf2	Kctd12	Nsmce2	S100a9	Tm4sf5
Apoa4	Cldn13	Fgf21	Klk1	Nts	S100g	Tmcc3
Apobec1	Cldn3	Fhad1	Klk1b11	Nup85	Saa3	Tmem19
Apoc2	Cldn4	Fnbp1	Klk7	Oasl1	Sat1	Tnf
Aqp3	Cldn7	Fscn1	Krt10	Ogfrl1	Sel1l3	Tnfrsf11b
Arc	Cnp	Gas7	Krt16	Oit1	Selm	Tnfrsf8
Asprv1	Cpeb4	Gbp4	Krt20	Ooep	Sema4a	Tnfsf9
Atp1a2	Crabp1	Gda	Krt77	Pcp4	Sephs2	Tnip1
Atp1b1	Crhbp	Gdf15	Krt79	Pgc	Serpinb12	Tnip3
Atp6v1c1	Csn2	Gjb2	Lad1	Pglyrp1	Serpinb1a	Tnmd
AU040320	Cst6	Gnat3	Laptm5	Pglyrp2	Serpinb2	Tnnt2
Avil	Ctrb1	Gnb3	Lcp1	Pigr	Serpinb9	Traf1
AW112010	Ctsh	Gnb4	Liph	Pip5k1b	Sgpp1	Trpm5
BC051142	Ctss	Gng13	Lrmp	Pla1a	Sh2d6	Ttn
Bcl2a1a	Ctsz	Gpa33	Lrrc42	Pla2g4a	Sh3tc2	Ubd
Bcl2l1	Cxcl2	Gramd4	Lsp1	Plagl1	Sirt1	Ucma
Blnk	Cyba	Grap	Ly6i	Plb1	Skint2	Unc93a
Bmp2k	Cybb	Grin2c	Lypd2	Pld1	Skint4	Utf1
Cadps	Cyp2a4	Gstm1	Lypd8	Plekha4	Skint9	Vdr
Calca	Cyp2a4|Cyp2a5	Gstt1	Lyz1	Pmaip1	Slc13a2	Vmo1
Calcb	Cystm1	Guca2b	Lyz1|Lyz2	Prap1	Slc43a3	Wfdc21
Casp1	Defb6	H2-Eb2	Malat1	Prg2	Slc4a8	
Casz1	Dgat2	H2-Oa	Marc1	Prg4	Slc5a8	
Ccdc184	Dio1	Hagh	Mctp1	Prokr2	Slc6a20a	
Ccdc88a	Dmkn	Hal	Mdm2	Psors1c2	Slco5a1	
Ccl17	Dmpk	Hamp	Me1	Ptgds	Smtnl1	
Ccl20	Dnase1l3	Hdc	Mep1a	Ptgs2	Sncg	
Ccl22	Dscaml1	Hopx	Mmp7	Pyy	Spink1	
Ccl27a	Eaf2	Icosl	Muc1	Rab25	Spink5	
Ccl5	Ebi3	Igf1	Muc13	Rac2	Spock3	

Supplementary Table 5 | Genes included in the cell cycle gene set.

Cenpf
Arl6ip1
Ccnb1
Tuba1c
Smc2
H2afx
Tubb5
Cenpa
Ccnb2
Ube2c
Nucks1
Dut
Hmgn2
Hmgb1
Mki67
Ccna2
Tmpo
Cks2
Tubb4b
Tuba1a
Hmgb2
Cenpe
Hist1h2ab
Ube2s
Ncapd2
H2afz
Dek
Ranbp1
Ran
Stmn1
Hnrnpa2b1
Ybx1
Pcna
Ptma
Rrm2
Ppia
Pcna-ps2
Hnrnpa3
Mcm6
Atp5g3
Txn1

Supplementary Table 6 | Genes driving the enrichment for GO categories.

Biological process

regulation of cell growth - 1

Kazald1	early progenitor genes
Clstn1	early progenitor genes
Ddr1	early progenitor genes
Igfbp2	early progenitor genes
Igfbp3	early progenitor genes
Igfbp5	early progenitor genes
Igfbp7	early progenitor genes
Htra1	postnatal progenitor genes
Cyr61	postnatal progenitor genes
Igfbp4	postnatal progenitor genes
Igfbp5	postnatal progenitor genes

response to mechanical stimulus - 2

Btg2
Fosb
Dcn
Igfbp2
Txnip
Thbs1
Tgfbr2

negative regulation of cell proliferation - 3

Bcl2
Btg2
Ar
Bmp4
Cyp1b1
Ddr1
Igfbp3
Nfib
Pmp22
Ptprz1
Spry1
Timp2
Tgfbr2

cell adhesion - 4

Boc
Col6a1
Col6a2
Cyr61

Dst
Emp2
Itga6
Itgb4
Lamb3
Tgfbi

positive regulation of epithelial cell proliferation - 5

Htra1
Glul
Gas1
Id1

antigen processing and presentation of exogenous peptide antigen via MHC class I, TAP- dependent - 6

Psma1
Psma5
Psma7
Psmb10
Psmb4
Psmb9
Psmb11

proteolysis involved in cellular protein catabolic process - 7

Ctsl
Psma1
Psma5
Psma7
Psmb10
Psmb4
Psmb9
Psmb11

lipid metabolic process - 8

Agpat3
Cds1
Oma1
Cpt1a
Etnk1
Fam213b
Fabp5
Fasn
Hadhb
Hpgd
Lpl
Lpcat2
Pla2g16
Pltp

Sgpl1
Scd2
Srebf1

immune response - 9

Oasl1
Blnk
Cd70
Sh2d6
Ctss
Ccl17
Ccl20
Ccl22
Ccl27a
Ccl5
Ccl6
Ccl9
Ccr7
Cxcl2
H2-Oa
H2-Eb2
Il12a
Il13
Il23a
Prg2
Prg4
Serpinb9
Tnfsf9
Tnfrsf11b
Tnfrsf8
Tnf

inflammatory response - 10

Elf3
S100a8
S100a9
Tnip1
Calca
Ccl17
Ccl20
Ccl22
Ccl5
Ccl6
Ccl9
Ccr7
Cxcl2
Chil1
Crhbp

Cyba
Cybb
Il13
Il23a
Krt16
Ncf1
Nos2
Ptgs2
Reg3g
Tnfrsf11b
Tnfrsf8
Tnf

chemotaxis - 11

Rac2
S100a8
S100a9
Ccl17
Ccl20
Ccl22
Ccl5
Ccl6
Ccl9
Ccr7
Cxcl2
Lsp1
Nup85

Supplementary Table 7 | Barcode IDs and cell numbers in different TEC compartments of E16.5 thymic epithelium.

barcode ID_E16.5	1	10	11	17	2	2_24
cT	272	7	3	9	75	1
mT	0	0	0	0	0	0
pp	32	0	1	1	15	0
ep	146	4	6	2	44	0

barcode ID_E16.5	20	22	23	3	3_4	32
cT	3	2	1	12	1	1
mT	0	0	0	0	0	0
pp	1	0	0	0	0	0
ep	6	0	1	1	1	1

barcode ID_E16.5	35	4	46	5	58	59
cT	5	13	2	50	1	15
mT	0	0	0	0	0	0
pp	1	3	1	7	0	1
ep	2	13	1	33	1	6

barcode ID_E16.5	59_60	6	62	63	64	65
cT	2	13	0	5	4	0
mT	0	0	0	0	0	0
pp	0	2	0	2	1	1
ep	1	10	1	7	4	0

barcode ID_E16.5	66_46	67	7	8	9	0
cT	4	6	8	0	5	53
mT	0	0	0	0	0	0
pp	0	0	3	0	1	9
ep	1	1	3	1	6	30

Supplementary Table 8 | Barcode IDs and cell numbers in different TEC compartments of P0 thymic epithelium.

barcode ID_P0	1	18	2	3	32	33
cT	313	11	9	0	12	1
mT	62	0	7	1	0	0
pp	28	0	7	1	0	0
ep	82	4	2	0	1	0

barcode ID_P0	34	35	4	41	5	53
cT	1	0	15	0	10	10
mT	0	1	2	0	20	0
pp	0	0	1	0	5	0
ep	0	0	2	0	4	2

barcode ID_P0	54	56	57	58	8	0
cT	7	0	0	1	13	242
mT	0	6	0	0	3	66
pp	1	3	0	0	0	32
ep	4	0	1	0	6	53

Supplementary Table 9 | Barcode IDs and cell numbers in different TEC compartments of P28 male thymic epithelium.

barcode ID_P28 male	1	100	101	102	105
cT	26	0	0	0	0
mT	174	1	0	1	0
pp	22	0	0	0	0
ep	237	0	1	0	1

barcode ID_P28 male	106	107	2	2_4	21
cT	0	0	9	0	0
mT	0	0	50	2	7
pp	0	0	9	0	1
ep	1	1	78	0	3

barcode ID_P28 male	28	3	34_2	4	41
cT	0	1	0	0	0
mT	3	3	4	7	0
pp	1	2	0	1	0
ep	4	3	4	14	1

barcode ID_P28 male	42	5	5_2	58	59
cT	0	7	1	0	0
mT	0	59	2	0	0
pp	0	6	0	0	0
ep	1	54	7	1	1

barcode ID_P28 male	6	68	68_2	68_5	7
cT	0	0	1	0	0
mT	3	0	7	5	3
pp	0	0	0	0	0
ep	4	2	2	3	5

barcode ID_P28 male	8	83	84	85	86
cT	0	0	0	1	0
mT	6	5	2	3	4
pp	2	0	1	0	0
ep	5	6	1	4	3

barcode ID_P28 male	87	88	89	90	90_5
cT	0	0	1	0	0
mT	2	4	1	2	0
pp	0	0	0	0	0
ep	4	3	6	3	1

barcode ID_P28 male	91	91_5	92	93	94_2
cT	0	1	0	0	1
mT	1	2	0	1	2

pp	0	0	0	0	0
ep	0	2	1	0	6

barcode ID_P28 male	95	95_7	96	96_8	97_5
cT	0	0	0	0	0
mT	0	6	5	0	0
pp	0	0	1	0	0
ep	1	3	1	2	1

barcode ID_P28 male	0
cT	28
mT	82
pp	5
ep	78

Supplementary Table 10 | Barcode IDs and cell numbers in different TEC compartments of P28_female_1 thymic epithelium.

barcode ID_P28 female 1	1	109	110	13	14
cT	15	0	1	1	0
mT	40	2	1	3	3
pp	8	1	1	1	0
ep	53	2	3	4	6

barcode ID_P28 female 1	16	19	2	21	25
cT	7	0	11	0	0
mT	0	4	13	1	1
pp	0	0	4	0	0
ep	0	2	12	1	0

barcode ID_P28 female 1	26	27	28	29	3_12
cT	0	0	0	1	0
mT	1	0	0	0	3
pp	0	0	0	0	0
ep	0	1	1	0	5

barcode ID_P28 female 1	4	41	5	7	0
cT	0	0	2	1	35
mT	0	1	7	1	9
pp	0	0	0	0	3
ep	1	0	8	1	11

barcode ID_P28 female 1	3_15	30	31
cT	1	1	1
mT	3	0	0
pp	0	0	0
ep	2	0	0

Supplementary Table 11 | Barcode IDs and cell numbers in different TEC compartments of P28_female_2 thymic epithelium.

barcode ID_P28 female 2	1	2	4	41	42
cT	52	0	0	0	2
mT	111	11	9	9	2
pp	20	1	1	1	0
ep	64	5	3	7	4

barcode ID_P28 female 2	43	44	45	48	5
cT	1	0	1	0	1
mT	4	4	2	0	6
pp	1	0	0	0	1
ep	2	2	2	1	2

barcode ID_P28 female 2	51	8	0
cT	0	1	48
mT	0	7	135
pp	0	0	18
ep	1	5	86

Supplementary Table 12 | Barcode IDs and cell numbers in different TEC compartments of 1 yr thymic epithelium.

barcode ID_1y	1	15	2	29	41	44
cT	19	1	9	4	0	1
mT	18	1	4	0	2	0
pp	80	1	22	8	15	1
ep	0	0	0	0	0	0

barcode ID_1y	5	68	69	70	71
cT	21	4	0	0	0
mT	28	0	0	0	0
pp	93	4	1	1	1
ep	0	0	0	0	0

barcode ID_1y	73	74	0
cT	1	7	13
mT	0	0	11
pp	2	3	41
ep	0	0	0

Supplementary Table 13 | Barcode IDs and cell numbers in different TEC compartments of P28_Fgf7_tg thymic epithelium.

barcode ID_Fgf7 tg P28	1	15	2	21	3
cT	308	1	1	2	4
mT	98	0	2	1	1
pp	482	0	10	4	1
ep	270	0	4	4	4

barcode ID_Fgf7 tg P28	4	43	45	5	5_74
cT	7	6	0	11	6
mT	3	1	0	1	0
pp	11	2	0	13	3
ep	2	3	1	12	1

barcode ID_Fgf7 tg P28	59	76	78	8	8_81
cT	0	5	5	4	2
mT	0	1	0	0	1
pp	1	8	5	12	2
ep	0	3	1	6	2

barcode ID_Fgf7 tg P28	80_45	81	0
cT	3	0	21
mT	1	0	5
pp	2	1	19
ep	1	0	13

Supplementary Table 14 | Barcode IDs and cell numbers in different TEC compartments of 1 yr_Fgf7_tg thymic epithelium.

barcode ID_Fgf7 tg 1y	1	111	112	112_2	112_4
cT	2	1	0	0	0
mT	9	1	1	0	0
pp	121	24	20	3	1
ep	0	0	0	0	0

barcode ID_Fgf7 tg 1y	113	114	115	116	117
cT	1	0	0	2	0
mT	1	1	1	0	1
pp	8	7	4	9	5
ep	0	0	0	0	0

barcode ID_Fgf7 tg 1y	118	118_2	119	119_4	120
cT	0	0	0	0	1
mT	0	0	0	0	1
pp	8	1	10	1	8
ep	0	0	0	0	0

barcode ID_Fgf7 tg 1y	121	122	122_2	123	124
cT	0	0	0	0	0
mT	3	0	0	0	0
pp	10	2	1	2	1
ep	0	0	0	0	0

barcode ID_Fgf7 tg 1y	125_2	126_36	130	131	2
cT	0	0	0	0	1
mT	0	0	0	0	10
pp	1	1	1	1	140
ep	0	0	0	0	0

barcode ID_Fgf7 tg 1y	2_111	2_129	2_4	2_8	28
cT	0	0	0	0	0
mT	0	0	1	0	0
pp	3	1	1	1	6
ep	0	0	0	0	0

barcode ID_Fgf7 tg 1y	28_36	3	35	36	36_111
cT	0	0	0	1	0
mT	1	0	0	3	3
pp	4	1	1	49	21
ep	0	0	0	0	0

barcode ID_Fgf7 tg 1y	36_2	36_46	36_8	4	46
cT	0	0	0	0	0
mT	0	0	0	4	0

pp		1	3	1	24	1
ep		0	0	0	0	0
barcode ID_Fgf7 tg 1y		5	5_2	5_36	5_4	5_7
cT		1	0	0	0	0
mT		3	1	0	0	0
pp		35	4	8	3	11
ep		0	0	0	0	0
barcode ID_Fgf7 tg 1y	5_8		59	59_2	65	7
cT		0	1	0	0	2
mT		0	2	0	0	1
pp		1	26	1	8	23
ep		0	0	0	0	0
barcode ID_Fgf7 tg 1y	7_2		7_36	76	8	0
cT		0	0	0	0	2
mT		0	0	0	3	5
pp		2	1	9	13	84
ep		0	0	0	0	0

Supplementary Table 15 | Genotyping information.

Transgene	Forward primer 5'-3'	Reverse primer 5'-3'	Amplicon size (bp)
Foxn1:Fgf7	CCGTGGCAGTTGGAATTGTGGC	ACATTTCCCCTCCGCTGTGTGTC	186
Foxn1:Fgfr2b	CTGGCTCACTGTCCTGCCCAA	AGCATCGGGGTGTCCGCTGT	324
hU6:sgRNA^{Hprt}	GGACTATCATATGCTTACCG	CGACTCGGTGCCACTTTTTC	171
Foxn1:eGFP	Xah163	L1_EGFP	325
Foxn1:Cre	TGCATGATCTCCGGTATTGA	CGTACTGACGGTGGGAGAAT	374
Rosa26LSLEYFP	AAAGTCGCTCTGAGTTGTTAT	GCGAAGAGTTTGTCCTCAACC	300 (*Rosa locus* flox/del)
	AAAGTCGCTCTGAGTTGTTAT	GGAGCGGGAGAAATGGATATG	600 (wt *Rosa locus*)
Foxn1:s-Fgfr2b	AAAGTCGCTCTGAGTTGTTAT	CAGACTTCTTCCCTGAAGAC	300
pLck:Cre	TGCATGATCTCCGGTATTGA	CGTACTGACGGTGGGAGAAT	
Rosa26LSLCas9EYFP	AAAGTCGCTCTGAGTTGTTAT	GGAGCGGGAGAAATGGATATG	600 (wt *Rosa locus*)
	CCACGACGGAGACTACAAGG	GGGCTCCGATCAGGTTCTTC	254 (*Cas9*)

Supplementary Table 16 | Antibodies and staining reagents used in this study.

**Antigen/Reagent**	**Clone**	**Conjugate**	**Supplier**	**Catalogue Number**	**dilution**
CD4	GK1.5	FITC	BioLegend	100406	1:1000
CD8a	53-6.7	FITC<br>APC	eBioscience	11-0081-82<br>17-0081-82	1:200<br>1:1000
CD31	MEC13.3	FITC	BioLegend	102506	1:300
CD45	30-F11	PE Cy7	BioLegend	103114	1:2000
EpCAM	G8.8	APC	BioLegend	118214	1:1000
Keratin 5	rabbit polyclonal	-	Covance	PRB-160P	1:500
Keratin 8	Troma-1	-	produced in house	-	1:200
Ly51	6C3	PE	eBioscience	12-5891-83	1:300
Streptavidin	-	Cy3	Jackson ImmunoResearch	016-160-084	1:1000
Streptavidin	-	eFluor450	eBioscience	48-4317-82	1:1000
UEA-1	-	FITC	Vector Laboratories	FL-1061-5	1:1000
Keratin 18	Ks18.04	biotin	PROGEN	61528	1:10
mCardinal (RFP)	rabbit polyclonal	-	ThermoFisher Scientific	R10367	1:500
MHC2	M5/114.15.2	FITC	BioLegend	107606	1:4000
rabbit IgG (H+L)	goat polyclonal	Alexa Fluor 633	Invitrogen Molecular Probes	A-21070	1:500
rat IgG (H+L)	donkey polyclonal	Cy3	Jackson ImmunoResearch	712-166-153	1:500
