# Supplementary material for: Developmental dynamics of two bipotent thymic epithelial progenitor types
Source: Nature. 2022 May 25;606(7912):165–71. doi: 10.1038/s41586-022-04752-8 (PMC9159946; doi:10.1038/s41586-022-04752-8)
Supplement: Supplementary file 2 — Reporting Summary [file 41586_2022_4752_MOESM2_ESM.pdf]

nature research

Corresponding author(s): Thomas Boehm
Last updated by author(s): 24/03/2022

Reporting Summary

Nature Research wishes to improve the reproducibility of the work that we publish. This form provides structure for consistency and transparency in reporting. For further information on Nature Research policies, see our Editorial Policies and the Editorial Policy Checklist.

Statistics

For all statistical analyses, confirm that the following items are present in the figure legend, table legend, main text, or Methods section.

n/a	Confirmed	
☐	☒	The exact sample size (n) for each experimental group/condition, given as a discrete number and unit of measurement
☐	☒	A statement on whether measurements were taken from distinct samples or whether the same sample was measured repeatedly
☐	☒	The statistical test(s) used AND whether they are one- or two-sided<br>*Only common tests should be described solely by name; describe more complex techniques in the Methods section.*
☒	☐	A description of all covariates tested
☐	☒	A description of any assumptions or corrections, such as tests of normality and adjustment for multiple comparisons
☐	☒	A full description of the statistical parameters including central tendency (e.g. means) or other basic estimates (e.g. regression coefficient) AND variation (e.g. standard deviation) or associated estimates of uncertainty (e.g. confidence intervals)
☐	☒	For null hypothesis testing, the test statistic (e.g. F, t, r) with confidence intervals, effect sizes, degrees of freedom and P value noted<br>*Give P values as exact values whenever suitable.*
☒	☐	For Bayesian analysis, information on the choice of priors and Markov chain Monte Carlo settings
☒	☐	For hierarchical and complex designs, identification of the appropriate level for tests and full reporting of outcomes
☐	☒	Estimates of effect sizes (e.g. Cohen's d, Pearson's r), indicating how they were calculated

Our web collection on statistics for biologists contains articles on many of the points above.

Software and code

Policy information about availability of computer code

Data collection	FACS Diva Software v8.0.2, Summit 5.5 (MoFlow)
Data analysis	FlowJo 9.3.1 for flow cytometric analyses, GraphPad Prism 9 for data presentation<br>For a description of software/codes used for the present study, see<br>J. S. Herman, Sagar, D. Grün, FateID infers cell fate bias in multipotent progenitors from single-cell RNA-seq data. Nat Methods 15, 379-386 (2018).<br>D. Grün, Revealing dynamics of gene expression variability in cell state space. Nat Methods 17, 45-49 (2020).

For manuscripts utilizing custom algorithms or software that are central to the research but not yet described in published literature, software must be made available to editors and reviewers. We strongly encourage code deposition in a community repository (e.g. GitHub). See the Nature Research guidelines for submitting code & software for further information.

Data

Policy information about availability of data

All manuscripts must include a data availability statement. This statement should provide the following information, where applicable:

- Accession codes, unique identifiers, or web links for publicly available datasets
- A list of figures that have associated raw data
- A description of any restrictions on data availability

The primary read files as well as expression count files for the single-cell RNA sequencing datasets reported in this paper are available to download from GEO (accession number: GSE106856). No restrictions apply.

Field-specific reporting

Please select the one below that is the best fit for your research. If you are not sure, read the appropriate sections before making your selection.

☒ Life sciences ☐ Behavioural & social sciences ☐ Ecological, evolutionary & environmental sciences

For a reference copy of the document with all sections, see nature.com/documents/nr-reporting-summary-flat.pdf

Life sciences study design

All studies must disclose on these points even when the disclosure is negative.

Sample size	The sample sizes for each experiment are indicated in the figure legends. Sample sizes were based on our experience and accepted practice in the respective fields, balancing statistic robustness, resource availability, and animal welfare. No statistical methods were used to predetermine sample size.
Data exclusions	No data were excluded.
Replication	The wildtype scRNA-seq data analyses were replicated 4 times. Experiments were performed independently and results of replicate experiments were in agreement. The presented findings were compared against data from public datasets where applicable. The results of several independent methods, e.g. flow cytometry, immunohistology, and transcriptome analyses of different transgenic mouse strains were in agreement.
Randomization	Provided the transgenic status, age, and sex of mice matched the experimental requirements, mice were randomly assigned to experimental groups.
Blinding	Blinding was not possible, as the thymus phenotype, i.e. the transgenic status of the respective mouse, is evident from flow cytometry or imaging analysis.

Reporting for specific materials, systems and methods

We require information from authors about some types of materials, experimental systems and methods used in many studies. Here, indicate whether each material, system or method listed is relevant to your study. If you are not sure if a list item applies to your research, read the appropriate section before selecting a response.

Materials & experimental systems

n/a	Involved in the study	
☐	☒	Antibodies
☒	☐	Eukaryotic cell lines
☒	☐	Palaeontology and archaeology
☐	☒	Animals and other organisms
☒	☐	Human research participants
☒	☐	Clinical data
☒	☐	Dual use research of concern

Methods

n/a	Involved in the study	
☒	☐	ChIP-seq
☐	☒	Flow cytometry
☒	☐	MRI-based neuroimaging

Antibodies

Antibodies used	α-CD4 (clone GK1.5; FITC conjugate; Cat#100406, BioLegend); α-CD8a (clone 53-6.7; FITC conjugate; Cat#11-0081-82, eBioscience); α-CD8a (clone 53-6.7; APC conjugate; Cat#17-0081-82, eBioscience); α-CD31 (clone MEC13.3; FITC conjugate; Cat#102506, BioLegend); α-CD45 (clone 30-F11; PE-Cy7 conjugate; Cat#103114, BioLegend); α-EpCAM (clone G8.8; APC conjugate; Cat#118214, BioLegend); α-Keratin5 (rabbit polyclonal; Cat#PRB-160P, Covance); α-Keratin-8 (clone Troma-1; produced in-house); α-Ly51 (clone 6C3; PE conjugate; Cat#12-5891-83, eBioscience); Streptavidin (eFluor450 conjugate; Cat#48-4317-82, eBioscience; Streptavidin (Cy3 conjugate; Cat#016-160-084, Jackson ImmunoResearch); UEA-1 (FITC conjugate; Cat# FL-1061-5, Vector Laboratories); Keratin 18 Ks18.04 (biotin-conjugate; Cat# 61528,PROGEN); mCardinal (RFP) rabbit polyclonal (Cat# R10367, ThermoFisher Scientific), MHC2 rat (M5/114.15.2; FITC conjugate; Cat#107606; BioLegend); rabbit IgG (H+L) (goat polyclonal; Alexa Fluor 633 conjugate; Cat#A-21070; Invitrogen Molecular Probes); rat IgG (H+L) (donkey polyclonal; Cy3 conjugate; Cat#712-166-153; Jackson ImmunoResearch). The dilutions used for each antibody are given in Supplementary Table 16.
Validation	see information associated with catalog numbers; https://www.biolegend.com/; https://www.thermofisher.com/; https://www.bioz.com/result/anti%20keratin%205/product/Covance; https://www.jacksonimmuno.com/; https://vectorlabs.com/

Animals and other organisms

Policy information about studies involving animals; ARRIVE guidelines recommended for reporting animal research

Laboratory animals	C57BL/6 mice are maintained in the Max Planck Institute of Immunobiology and Epigenetics. Foxn1:eGFP, Foxn1:Cre ,Foxn1:mCardinal, Rosa26LSLEYFP, Foxn1:s-Fgfr2IIIb, and Rosa26LSLCas9EYFP transgenic mice were described earlier. The Foxn1:Fgf7 transgene was created by inserting a cDNA fragment corresponding to nucleotides 347 to 934 in NM_008008 as a NotI fragment into pAHB14; in some old female Foxn1:Fgf7 transgenic mice (FVB/N-tg(Foxn1-Fgf7)1Tbo/Mpie), the two thymic lobes were asymmetric in size and shape; these mice were not included in our analysis. The Foxn1:Fgfr2IIIb transgene was created by inserting a cDNA fragment corresponding to nucleotides 1214 to 3366 in NM_201601.2 as a NotI fragment into pAHB14 and used to generate transgenic mice (FVB/N-tg(Foxn1-Fgfr2)1Tbo/Mpie). The hU6:sgRNAHprt transgene was cloned as a NotI fragment into the Bluescript vector and consists of the human U6 promotor (nucleotides 1-264 in JN255693), followed by the mouse Hprt target sequence (nucleotides 255-274 in J00423), and the sgRNA backbone (nucleotides 218-139 [reverse complement] in Addgene plasmid #42250), followed by a short 3´-sequence (T6G2A2); for injection into fertilized eggs, the construct was linearized with SacI. Transgenic mice were generated on an FVB/N background (FVB/N-tg(hU6-sgRNA-Hprt)1Tbo/Mpie) and subsequently backcrossed to a C57BL/6J background. For timed matings, the day of plug detection was designated as E0.5. Genotyping information is summarized in Supplementary Table 15. Mice were selected for analysis according to transgenic status, age, and sex according to experimental design. Mice were kept in the animal facility of the Max Planck Institute of Immunobiology and Epigenetics under specific pathogen-free conditions.
Wild animals	No wild animals were used in this study.
Field-collected samples	No field-collected animals were used in this study.
Ethics oversight	All animal experiments were performed in accordance with the relevant guidelines and regulations, approved by the review committee of the Max Planck Institute of Immunobiology and Epigenetics and the Regierungspräsidium Freiburg, Germany (licence 35-9185.81/G-12/85; 35-9185.81/G-16/67).

Note that full information on the approval of the study protocol must also be provided in the manuscript.

Flow Cytometry

Plots

Confirm that:

☒ The axis labels state the marker and fluorochrome used (e.g. CD4-FITC).

☒ The axis scales are clearly visible. Include numbers along axes only for bottom left plot of group (a 'group' is an analysis of identical markers).

☐ All plots are contour plots with outliers or pseudocolor plots.

☒ A numerical value for number of cells or percentage (with statistics) is provided.

Methodology

Sample preparation	To generate single cell suspensions for analytical and preparative flow cytometry of TECs, the procedures described by Nagakubo et al., Sci. Rep. 2017 and Rode et al., J. Immunol. 2015 were followed. Note that the enzymatic cocktail required to liberate thymic epithelial cells destroys the extracellular domains of CD4 and CD8 surface markers (but not that of the CD45 molecule); hence, when analysis of thymocyte subsets was desired, thymocyte suspensions were prepared in parallel by mechanical liberation, achieved by gently pressing thymic lobes through 40 µm sieves. To isolate thymic mesenchymal and endothelial cells, the cell suspension of total thymocytes was depleted of CD45+ cells; the EpCAM–CD45– cell population was stained with Ly51 and CD31 to purify EpCAM–CD31–Ly51+ mesenchymal and EpCAM–CD31+Ly51– endothelial cells.
Instrument	BD Fortessa II; MoFlow; both from Dako Cytomation-Beckman Coulter
Software	BD FACSDiva for data collection, Summit for sorting TECs, FlowJo for data analysis, statistical analysis with Graphpad Prism
Cell population abundance	Purity was determined by running a purity check of the sorted populations after the sort was completed.
Gating strategy	All samples were initially gated using forward and side scatter to identify events corresponding to cells, doublets are excluded by gating on single cells using forward scatter height vs. area, alive cells were selected by negativity for the viability dye Fluoro Gold, the follow gating steps are according to the marker genes described in the manuscript; see Extended Data Figure 7a.

☒ Tick this box to confirm that a figure exemplifying the gating strategy is provided in the Supplementary Information.
